# Avian influenza viruses suppress innate immunity by inducing *trans*-transcriptional readthrough *via* SSU72

**DOI:** 10.1038/s41423-022-00843-8

**Published:** 2022-03-24

**Authors:** Yan Zhao, Fengming Huang, Zhen Zou, Yuhai Bi, Yang Yang, Cong Zhang, Qiang Liu, Daozhen Shang, Yiwu Yan, Xiangwu Ju, Song Mei, Peng Xie, Xiao Li, Mingyao Tian, Shuguang Tan, Huijun Lu, Zongsheng Han, Kangtai Liu, Yuqing Zhang, Junbo Liang, Zhu Liang, Qingchao Zhang, Jiahui Chang, William J. Liu, Cong Feng, Tanshi Li, Michael Q. Zhang, Xiaoyue Wang, George F. Gao, Yingxia Liu, Ningyi Jin, Chengyu Jiang

**Affiliations:** 1 State Key Laboratory of Medical Molecular Biology, Institute of Basic Medical Sciences, Chinese Academy of Medical Sciences, Department of Biochemistry, Peking Union Medical College, Beijing, 100005 China; 2 Shenzhen Key Laboratory of Pathogen and Immunity, State Key Discipline of Infectious Disease, Second Hospital Affiliated with Southern University of Science and Technology, Shenzhen Third People’s Hospital, Shenzhen, Guangdong 518112 China; 3 CAS Key Laboratory of Pathogenic Microbiology and Immunology, Institute of Microbiology, Center for Influenza Research and Early-warning (CASCIRE), CAS-TWAS Center of Excellence for Emerging Infectious Diseases (CEEID), Chinese Academy of Sciences, Beijing, 100101 China; 4 School of Life Science, University of Science and Technology of China, Hefei, Anhui 230027 China; 5 Department of Automation, Tsinghua University, Beijing, 100084 China; 6 Genetic Engineering Laboratory, Institute of Military Veterinary, Academy of Military Medical Sciences, Changchun, Jilin 130062 China; 7 State Key Laboratory of Biotherapy/Collaborative Innovation Center for Biotherapy, West China Hospital, Sichuan University, Chengdu, Sichuan 610000 China; 8 State Key Laboratory of Medical Molecular Biology, Department of Biochemistry and Center for Bioinformatics, Institute of Basic Medical Sciences, Chinese Academy of Medical Sciences, School of Basic Medicine, Peking Union Medical College, Beijing, 100005 China; 9 National Institute for Viral Disease Control and Prevention, Chinese Centers for Disease Control and Prevention, Beijing, 102206 China; 10 Department of Emergency, General Hospital of the PLA, Haidian District, Beijing, 100853 China; 11 Department of Biological Sciences and Center for Systems Biology, The University of Texas at Dallas, Richardson, TX USA

**Keywords:** AIV infection, TRT, SSU72, NS1, Immune escape, Immune evasion, Infection

## Abstract

Innate immunity plays critical antiviral roles. The highly virulent avian influenza viruses (AIVs) H5N1, H7N9, and H5N6 can better escape host innate immune responses than the less virulent seasonal H1N1 virus. Here, we report a mechanism by which transcriptional readthrough (TRT)-mediated suppression of innate immunity occurs post AIV infection. By using cell lines, mouse lungs, and patient PBMCs, we showed that genes on the complementary strand (“*trans*” genes) influenced by TRT were involved in the disruption of host antiviral responses during AIV infection. The *trans*-TRT enhanced viral lethality, and TRT abolishment increased cell viability and STAT1/2 expression. The viral NS1 protein directly bound to SSU72, and degradation of SSU72 induced TRT. SSU72 overexpression reduced TRT and alleviated mouse lung injury. Our results suggest that AIVs infection induce TRT by reducing SSU72 expression, thereby impairing host immune responses, a molecular mechanism acting through the NS1-SSU72-*trans*-TRT-STAT1/2 axis. Thus, restoration of SSU72 expression might be a potential strategy for preventing AIV pandemics.

## introduction

Avian influenza viruses (AIVs) cause sporadic outbreaks and may result in uncontrolled pandemics because treatment options are extremely limited [1–4]. The mortality rate in patients infected with AIVs such as H5N1, H7N9, and H5N6 is higher than that in those infected with seasonal influenza viruses [5, 6]. Understanding the precise mechanisms by which influenza viruses infect their hosts and how the host immune system responds to these viruses is important for developing adequate preventive strategies for pandemics.

Although innate immune responses play critical antiviral roles, viruses can escape these responses, leading to high mortality rates [7]. Influenza viruses suppress antiviral innate immune responses, particularly the interferon (IFN)-β–Janus kinase (JAK)–signal transducer and activator of transcription (STAT) signaling pathway, *via* translation of nonstructural protein 1 (NS1), a multifunctional immunosuppressive factor [8]. NS1 targets JAK/STAT by reducing IFN signaling [9], binds to cleavage and polyadenylation specificity factor 30 (CPSF30), and inhibits mRNA cleavage at the poly(A) binding site [10]. NS1 interacts with cellular mRNA and the spliceosome component U6 snRNA, which inhibits the export of cellular RNAs from the nucleus [11]; inhibits the cellular antiviral 2′-5′-oligoadenylate synthetase–RNase L pathway, RNA-dependent protein kinase pathway, and retinoic acid-induced gene I expression [9, 12, 13]; and upregulates the JAK/STAT inhibitors SOCS1 and SOCS3 [14, 15]. NS1 could be the factor underlying viral virulence, as it can potentially inhibit the polyadenylation of cellular pre-mRNAs [16].

Herein, we report a novel mechanism by which lethal AIVs might suppress innate immunity, namely, transcriptional readthrough (TRT)-mediated repression of innate immunity. In TRT, transcription does not faithfully stop at the transcription termination site (TTS) but continues, resulting in the synthesis of RNA transcripts of up to dozens of kilobases [17]. Many studies have suggested that the RNA polymerase II C-terminal domain phosphatase SSU72 is involved in transcription termination [18–20]; SSU72 was first discovered in a screen for suppressors of transcription factor IIB in yeast [21] and was shown to be essential for transcription termination of small nucleolar RNAs and specific mRNAs in yeast [22]. More recently, ChIP-seq and GRO-seq analyses revealed that SSU72 has a minor peak in the terminator region in human embryonic stem cells and thus has a modest effect on transcription termination [23]. In eukaryotic cells, transcription is precisely regulated through cleavage and poly(A) signals [24]. Abnormal TRT has been reported in HIV-1 [25], HSV-1 [26], and Leishmania infections [27] as well as in renal carcinoma [28] and under osmotic stress [29, 30]; however, the underlying mechanism is not yet known.

Recent studies have revealed that the influenza A virus NS1 protein induces global transcriptional defects at the 3′ ends of genes and causes global TRT, which can dysregulate host transcription [31]. Moreover, NS1-induced TRT may alter the three-dimensional organization of downstream chromatin [32]. Disruption of transcription termination was shown to be widespread and continuous and to extend from the gene coding sequence. The presence of RNA polymerase II in the TRT region was shown to influence spatial chromatin interactions, and TRT was shown to be involved in cohesion-mediated interactions of chromatin with gene bodies [32]. Here, we explored the functions of TRT in innate immune responses and immune escape upon AIV infection.

## Material and methods

### Ethics statement

All procedures in studies involving human participants were performed in accordance with the ethical standards of the institutional and/or national research committee and with the 1964 Declaration of Helsinki and its later amendments or comparable ethical standards. The study was approved by the Ethics Committees, and verbal informed consent was obtained from all patients or family members of patients.

All animal studies were conducted following the National Guidelines for the Care of Laboratory Animals and performed in accordance with institutional regulations and with the approval of experimental protocols by the Institutional Animal Care and Use Committee of the Institute of Military Veterinary, Academy of Military Medical Sciences.

### Cell lines and cell culture

The human lung adenocarcinoma cell lines A549 and H1650, human cervical adenocarcinoma cell line HeLa, and human embryonic kidney cell lines HEK293 and HEK293T were purchased from the American Type Culture Collection (ATCC; Rockville, MD, USA). Adult human pulmonary fibroblast (HPF-a) cells were purchased from ScienCell Research Laboratories (ScienCell Research Laboratories, Inc., Carlsbad, CA, USA). Madin–Darby canine kidney (MDCK) cells, the THP-1 human acute monocytic leukemia cells, and Jurkat human T lymphocyte cells were purchased from the Peking Union Medical College Cell Culture Center (Beijing, China).

A549 cells were cultured in Ham’s F-12 medium (HyClone Laboratories, Inc., South Logan, UT, USA); MDCK, H1650, HeLa, HEK293, and HEK293T cells were cultured in Dulbecco’s modified Eagle’s medium (DMEM; Gibco, Thermo Fisher Scientific, Inc., Waltham, MA, USA); peripheral blood mononuclear cells (PBMCs), THP-1, and Jurkat cells were cultured in RPMI-1640 medium (HyClone Laboratories; cat. no. SH30027.01); and HPF-a cells were cultured in fibroblast medium (ScienCell Research Laboratories; cat. no. 2301). Ham’s F-12 medium, DMEM, and RPMI-1640 medium were supplemented with 10% fetal bovine serum (FBS), penicillin (100 U/mL), and streptomycin (100 mg/mL). Fibroblast medium was supplemented with 10 mL of FBS, 5 mL of fibroblast growth supplement, penicillin (100 U/mL), and streptomycin (100 mg/mL). All cell types were cultured in a 37 °C incubator in 5% CO_2_. Poly-l-lysine-coated culture vessels (2 μg/cm^2^) were used to culture HPF-a cells.

### Viruses

The A/New Caledonia/20/1999(H1N1) influenza virus and A/chicken/Jilin/9/2004(H5N1) influenza virus were obtained from the Institute of Military Veterinary Medicine, Academy of Military Medical Sciences. The A/Beijing/CAS0001/2009(H1N1), A/Beijing/CAS0001/2007(H3N2), A/Anhui/1/2005 (H5N1), A/Shenzhen/Th002/2016(H5N6), and A/Anhui/1/2013(H7N9) viruses were obtained from CAS Center for Influenza Research and Early-warning (CASCIRE). All live virus experiments were performed at biosafety level 3 facilities following governmental and institutional guidelines. Viruses were propagated by inoculation into 10- to 11-day-old specific-pathogen-free (SPF) embryonated chicken eggs *via* the allantoic route. Allantoic fluid (AF) was collected from the eggs after 2-3 days of incubation at 37 °C; the viral titer was determined in MDCK cells by using the Reed–Muench method and is expressed as TCID_50_ per milliliter as previously described [33]. The titrated viruses were stored at −80 °C.

### Separation of PBMCs

Blood samples were collected from patients with pneumonia and healthy controls at Peking Union Medical College Hospital. Blood samples from H1N1-infected patients were collected at the General Hospital of the PLA. In addition, blood was also obtained from patients admitted to Shenzhen Third People’s Hospital with avian influenza (H5N6/H7N9) infection (detailed patient information is shown in Supplementary Table 2). Four samples from H7N9-infected patients and eleven samples from H7N9-infected patients were collected during the first and second weeks of disease onset, respectively. Eleven samples from H7N9-infected patients were collected more than two weeks after disease onset. Written informed consent was obtained from all individuals.

Blood samples were collected into EDTA anticoagulant tubes, and PBMCs were isolated using Lymphoprep (STEMCELL Technologies Inc., Kent, WA, USA) according to the manufacturer’s instructions (STEMCELL Technologies; cat. no. 07851).

### RNA-seq and data analysis

Total RNA was extracted from cells or tissues by using Invitrogen TRIzol Reagent (Life Technologies Corporation, Carlsbad, CA, USA). RNA libraries were constructed and sequenced on the Illumina HiSeq 2500 platform (PE125). All RNA-seq analyses were performed by strand-specific transcriptome sequencing with the deoxy-UTP (dUTP) strand marking protocol.

We used FastQC (version 0.11.2) to control the read quality of the RNA-seq data. Bowtie2 (version 2.1.0) and TopHat2 (version 2.0.11) were used to align strand-specific paired-end RNA-seq reads from human cell lines, PBMCs, and tissue samples from patients infected with seasonal H1N1 or avian-origin H7N9/H5N6 influenza A virus to the human genome (version hg19, http://hgdownload.cse.ucsc.edu/downloads.html). Strand-specific paired-end RNA-seq reads from mouse tissue samples were mapped to the mouse genome (version mmc10, http://hgdownload.cse.ucsc.edu/downloads.html). Influenza A virus genomic coding sequences (ftp://ftp.ncbi.nih.gov/genomes/INFLUENZA/) encoding neuraminidase (NA) and hemagglutinin (HA) were used to confirm viral infection in cell lines and in mouse and human tissue samples. Cufflinks (version 2.2.1) was used to assemble transcription units and calculate gene expression levels in fragments per kilobase of exon model per million reads mapped (FPKM). We used Cuffmerge (version 2.2.1) and Cuffdiff (version 2.2.1) to compare the FPKM levels between samples.

### TRT definition

TRT genes are defined as genes with abnormal transcription in which the abnormal transcription signal has passed the TTS. We defined TRT-influenced genes as the nearest downstream genes influenced by TRT genes.

TRT can affect the expression of the nearest downstream genes *via* two mechanisms: affecting the TRT-influenced gene encoded on the same DNA strand (*cis*-TRT) or that encoded on the complementary DNA strand (*trans*-TRT).

### Calculation of TRT gene expression

We filtered 11,784 *cis*-gene pairs and 5052 *trans*-gene pairs from the human genome.

The standard for *cis*-TRT genes was calculated as follows:

1. The *cis*-TRT gene expression FPKM value was greater than 2.
2. The fold change (FC) in the expression level of the *cis*-TRT region was greater than 5 (upregulated) relative to the control group.
  FC in the expression of a *cis*-TRT region = *V*_*c*_(*X*_*j*_^*i*^)/*V*_*c*_(*X*_*0*_^*i*^),
  *i* = 1, 2, 3,…, *n*_*c*_ (number of *cis-*genes)
  *j* = 1, 2, 3,…, *m* (number of genes in the test group)
  where *V*_*c*_(*X*_*j*_^*i*^) represents the expression level of the *cis*-TRT region of a particular gene *X* in one of the test groups and *V*_*c*_(*X*_*0*_^*i*^) represents the expression level of the *cis*-TRT region of a particular gene *X* in the control group.

The standards for *trans*-TRT genes calculated from *trans*-gene pairs were similar to those for *cis*-TRT genes, as follows:

1. The *trans*-TRT gene expression FPKM value was greater than 2.
2. The FC in the expression level of the *trans*-TRT region was greater than 5 (upregulated) relative to that in the control group.
  FC in the expression of a *trans*-TRT region = *V*_*t*_(*Y*_*j*_^*k*^)/*V*_*t*_(*Y*_*0*_^*k*^),
  *k* = 1, 2, 3,…, *n*_*t*_ (number of *trans*-genes)
  *j* = 1, 2, 3,…, *m* (number of genes in the test group)
  where *V*_*t*_(*Y*_*j*_^*k*^) represents the expression level of the *trans*-TRT region of a particular gene *Y* in one of the test groups, and *V*_*t*_(*Y*_*0*_^*k*^) represents the expression level of the *trans*-TRT region of a particular gene *Y* in the control group.

### Calculation of TRT region expression

*Cis*-TRT region expression levels were calculated using the following formula:

*V*_*c*_(*X*^*i*^) = *R*_*c*_(*X*^*i*^)/(*T*_*c*_ × *L*_*c*_(*X*^*i*^)), *i* = 1, 2, 3,…, *n*_*c*_ (number of *cis-*genes) where *V*_*c*_(*X*^*i*^) represents the expression level of the *cis*-TRT region of a particular gene *X*; *R*_*c*_(*X*^*i*^) represents the RNA-seq read count mapped to the *cis*-TRT region; *T*_*c*_ represents the total RNA-seq read count (in millions) mapped to all 11,784 *cis*-gene pairs and *cis*-TRT regions; and *L*_*c*_(*X*^*i*^) denotes the length (in kb) of the *cis*-TRT region, which extends from the TTS of the *cis*-TRT gene to the TTS of the nearest *cis*-TRT-influenced gene on the same DNA strand.

The following formula was used to calculate *trans*-TRT region expression levels:

*V*_*t*_(*Y*^*i*^) = *R*_*t*_ (*Y*^*k*^)/(*T*_*t*_ × *L*_*t*_(*Y*^*k*^)), *k* = 1, 2, 3,…, *n*_*t*_ (number of *trans-*genes) where *V*_*t*_(*Y*^*k*^) represents the expression level of the *trans*-TRT region of a particular gene *Y*; *R*_*t*_(*Y*^*k*^) represents the RNA-seq read count mapped to the particular *trans*-TRT region; *T*_*t*_ represents the total RNA-seq read count (in millions) mapped to all 5052 *trans-*gene pairs and *trans*-TRT regions; and *L*_*t*_(*Y*^*k*^) denotes the length (in kb) of the *trans*-TRT region, which starts at the TTS of the *trans*-TRT gene and extends to the TTS of the nearest *trans*-TRT-influenced gene on the complementary DNA strand.

### Calculation TRT-influenced gene expression

We selected *cis*-TRT-influenced genes from the *cis*-gene pairs based on the following criteria:

1. The expression level of the *cis*-TRT-influenced gene in the control group was greater than 2.
2. The expression of the *cis*-TRT-influenced gene was downregulated with a FC of >1.5 relative to that in the control group.
  FC in the expression of a *cis*-TRT-influenced gene = *F*_*c*_(*P*_*0*_^*i*^)/*F*_*c*_(*P*_*j*_^*i*^),
  *i* = 1, 2, 3,…, *n*_*c*_ (number of *cis-*genes)
  *j* = 1, 2, 3,…, *m* (number of genes in the test group)
  where *F*_*c*_(*P*_*j*_^*i*^) represents the expression level of a *cis*-TRT-influenced gene of a particular gene *P* in one of the test groups, and *F*_*c*_(*P*_*0*_^*i*^) represents the expression level of the *cis*-TRT-influenced gene of a particular gene *P* in the control group.
3. The expression of the *cis*-TRT region was upregulated with a FC of >5 relative to that in the control group. The same criteria used for *cis*-TRT genes were used.

We used the following criteria to select *trans*-TRT-influenced genes from the *trans*-gene pairs:

1. The expression level of the *trans*-TRT-influenced gene in the control group was greater than 2.
2. The expression level of the *trans*-TRT-influenced gene was downregulated with a FC of >1.5 relative to that in the control group.
  FC in the expression of a *trans*-TRT-influenced gene = *F*_*t*_(*Q*_*0*_^*k*^)/*F*_*t*_(*Q*_*j*_^*k*^),
  *k* = 1, 2, 3,…, *n*_*t*_ (number of *trans-*genes)
  *j* = 1, 2, 3,…, *m* (number of genes in the test group)
  where *F*_*t*_(Q_*j*_^*k*^) represents the expression level of a *trans*-TRT-influenced gene of a particular gene *Q* in one of the test groups, and *F*_t_(*Q*_*0*_^*k*^) represents the expression level of a *trans*-TRT-influenced gene of a particular gene *Q* in the control group.
3. The expression of the *trans*-TRT region was upregulated with a FC of >5 relative to that in the control group. The same criteria used for *trans*-TRT genes were used.

### Analysis of gene profiles and RNA-seq coverage

We used BEDtools (version 2.23.0) to analyze gene profiles. We screened for genes without genes on the complementary DNA strand to identify downregulated differentially expressed genes (DEGs) (FC > 2) between the virus-infected and control groups. We divided the gene body (the region between the transcription start site (TSS) and TTS) of each DEG into 60 equally sized bins and then added the flanking antisense strand regions 4 kb upstream of the TSS and 4 kb downstream of the TTS in 100-bp windows. The normalized expression levels in each bin or window were calculated using Python (version 2.7.3, https://www.python.org/) and its default package NumPy. For the *trans*-gene pairs *GLS–STAT1* and *IL23A–STAT2*, RNA expression levels in the gene bodies, intergenic regions, and gene flanking regions upstream of the TSSs were combined into 50-bp windows.

### Gene functional enrichment analysis

We used MetaCore (Thomson Reuters, Philadelphia, PA, USA) software for functional and pathway enrichment analyses of TRT-influenced genes. Two-tailed *P* values and Benjamini–Hochberg-adjusted *P* values of < 0.05 were considered statistically significant.

### PTM analysis

A549 cells were treated with the A/chicken/Jilin/9/2004 (H5N1) virus at a multiplicity of infection (MOI) of 4 or with an equal volume of vehicle for 18 h and were then washed with phosphate-buffered saline (PBS). Disulfide bonds in equal amounts of protein lysate (10 mg/sample) were reduced by adding dithiothreitol (DTT) to a final concentration of 4.5 mM. Each sample was then mixed in the well and was placed in a 55 °C incubator for 30 min. The solution was briefly cooled on ice to room temperature, and iodoacetamide solution (100 mM, one-tenth volume) was then added to a final concentration of 10 mM. The sample was sufficiently mixed, incubated for 15 min at room temperature in the dark, and diluted 4-fold with 20 mM HEPES buffer (pH 8.0) to a final urea concentration of approximately 2 M. Subsequently, a one-hundredth volume of a 1 mg/mL trypsin-TPCK (Worthington, LS003744) stock solution in 1 mM HCl was added, and the mixture was digested overnight at RT with mixing. After digestion, a one-twentieth volume of 20% trifluoroacetic acid (TFA) was added to the digested sample to a final concentration of 1%. The pH was checked by spotting a small amount of the peptide sample onto a pH strip to ensure that the pH was less than 3. After acidification, a precipitate was allowed to form by maintaining the sample on ice for 15 min. The acidified peptide solution was then centrifuged at 1780 × *g* for 15 min at room temperature. The peptides in the digested sample were cleaned by passing the acidified sample through a C18 Sep-Pak cartridge (WAT051910; Waters) and eluting with 10 mL of 40% acetonitrile in 0.1% TFA. The eluted peptide solution was frozen overnight at –80 °C and subjected to lyophilization for 2 days.

An affinity matrix was prepared by incubating a ubiquitin branch motif antibody (Cell Signaling Technology; cat. no. 3925) with 30 µL of protein A-agarose beads (Roche Inc, Little Falls, NJ, USA). The samples were washed four times with 1× PBS after antibody binding. The lyophilized peptides were dissolved in 1.4 mL of 1× IAP buffer [50 mM MOPS, 10 mM sodium phosphate, and 50 mM sodium chloride (pH 7.2)]. The peptide solution was cleared by centrifugation at 10,000 × *g* for 5 min at 4 °C. The antibody beads were mixed with the peptide solution, and the mixture was incubated at 4 °C for 2 h. After immunoprecipitation, the supernatant was removed, and the beads were washed three times with 1 mL of 1× IAP buffer and three times with 1 mL of HPLC-grade water. The enriched peptides were eluted from the antibody beads by incubation in 50 µL of 0.15% TFA for 10 min at room temperature with gentle mixing; the elution step was then repeated, and the eluents were combined.

The enriched peptides were cleaned in a StageTip made in-house by placing two layers of C18 Empore^TM^ material into a 10 µL pipette tip. The StageTip was equilibrated by passing 50 µL of 50% acetonitrile (ACN) in 0.1% TFA, followed by two passages of 50 µL of 0.1% TFA, through the tip by centrifuging the tip at 1500 × *g* for 1 min. The eluted peptide solution was passed through the StageTip by two rounds of centrifugation at 1500 × *g* for 2 min each, and the StageTip was then washed twice in 50 µL of 0.1% TFA by centrifugation at 1500 × *g* for 1 min each. The peptides were eluted from the StageTip by passing 10 µL of 40% ACN in 0.1% TFA through the tip by two rounds of centrifugation at 750 × *g* for 1 min each. The eluent was then dried under vacuum.

Each sample was dissolved in 12 µL of 0.125% formic acid and separated on a reverse-phase C18 column (75 µm inner diameter × 10 cm) packed into a PicoTip emitter (approximately 8 µm inner diameter) with Magic C18 AQ (100 Å × 5 µm; New Objective). Each sample was split, and analytical replicate injections were run to increase the number of identifications and provide metrics for analytical reproducibility of the method. A standard peptide mix (MassPREP^TM^ Protein Digestion Standard Mix 1; Waters) was spiked into each sample (total amount, 100 fmol; 33 fmol per injection) before LC–MS/MS analysis on an Orbitrap Elite mass spectrometer coupled to an Easy-nLC 1000 system. The peptides in each sample were separated with a linear gradient from 2% to 32% ACN over 90 min, and both MS and MS/MS data were acquired in centroid mode. For precursor analysis, the Orbitrap resolution was set to 70,000 with an AGC target of 1e [6] and a scan range of 300 to 1500 m/z. The top 20 precursors were selected for MS/MS analysis during each duty cycle with a normalized collision energy of 35.

### Generation and identification of recombinant viruses

Recombinant viruses were generated by reverse genetics, as previouse study [34]. In brief, eight gene segments from an A/Puerto Rico/8/1934(H1N1) (PR8) or PR8 backbone with the NS sequence from the A/New Caledonia/20/1999(H1N1) virus or A/chicken/Jilin/9/2004(H5N1) virus were individually inserted into dual-promoter plasmids. MDCK and HEK293T cells were then cocultured at a ratio of 1:5 and transfected with 0.5 µg of each of the plasmids along with 10 µL of Lipofectamine 2000 (Thermo Fisher Scientific; cat. no. 11668019) in 1 mL of Opti-MEM (Invitrogen). After incubation for 6 h at 37 °C, the transfection mixture was removed, and 1 mL of Opti-MEM containing 2 µg/mL tosylsulfonyl phenylalanyl chloromethyl ketone-treated trypsin (Worthington Biochemical Corporation, USA) was added. After 48 h, the cell supernatant was inoculated into 9-day-old SPF chicken embryos to produce stock viruses. The rescued viruses were named as PR8 witld type (wt), PR8+H1N1-NS1, and PR8+H5N1-NS1, respectively. Each viral segment was amplified by quantitative reverse transcription PCR (RT–qPCR) and sequenced to confirm the identity of the virus.

### Viral infection

In all experiments, cells were infected with virus at an MOI of 4. Controls were treated with an equal volume of AF. A549 cells were seeded into 12-well plates at a density of 3 × 10^5^ cells per well. The cells were infected with the A/New Caledonia/20/1999(H1N1), A/chicken/Jilin/9/2004(H5N1), A/Beijing/CAS0001/2009(H1N1), A/Beijing/CAS0001/2007(H3N2), A/Anhui/1/2005(H5N1), A/Shenzhen/Th002/2016(H5N6), and A/Anhui/1/2013(H7N9) viruses and the following three recombinant viruses: the PR8 wt, the PR8+H1N1-NS1, and the PR8+H5N1-NS1.

H1650, HEK293, HEK293T, HPF-a, and HeLa cells were collected 24 h after infection with A/New Caledonia/20/1999(H1N1) or A/chicken/Jilin/9/2004(H5N1), whereas THP-1 and Jurkat cells and PBMCs were collected 24 h after infection with A/New Caledonia/20/1999(H1N1) or A/chicken/Jilin/9/2004(H5N1).

### Plasmids

Ten protein-coding fragments of the H5N1 virus (*PB1, PB2, PA, HA, NA, M1, M2, NS1, NS2*, and *NP*) were optimized for cDNA synthesis by using high-frequency human codons as previously described [35]. Each of these fragments was then fused to a Flag tag at the C-terminus and inserted into the *Peak*13 vector (provided by B. Seed, Harvard Medical School, Boston, MA, USA). The protein-coding fragments of the A/New Caledonia/20/1999(H1N1) virus were constructed using the same method.

Full-length cDNAs encoding human *SSU72* (NCBI Reference Sequence: NM_014188.2) were amplified by PCR from a cDNA library generated from A549 cells. The SSU72-Myc plasmid contained the full-length coding sequence of *SSU72* without a stop codon and the Myc-tag sequence inserted into the *Peak*13 vector. The NS1-green fluorescent protein (GFP) plasmid contained the protein-coding fragments of H5N1 NS1 fused to GFP, which was also inserted into the *Peak*13 vector. All gene products were verified by DNA sequencing (Invitrogen, Beijing, China).

The psPAX2 (Addgene, Watertown, MA, USA; cat. no. 12260), pMD2.G (Addgene; cat. no. 12259), lentiGuide-Puro (Addgene; cat. no. 52963), pHR-SFFV-dCas9-BFP-KRAB (Addgene; cat. no. 46911), and lenti-dCAS-VP64_Blast (SAM) (Addgene; cat. no. 6142) plasmids were purchased from Addgene.

### *SSU72* overexpression and knockdown

For *SSU72* overexpression, A549 cells were transfected with a control plasmid or a plasmid encoding human *SSU72* by using X-tremeGENE HP DNA Transfection Reagent (Roche; cat. no. 6366236001) for 60 h according to the manufacturer’s instructions and were then infected with the A/chicken/Jilin/9/2004(H5N1) virus at an MOI of 4 for 48 h or treated with an equal volume of vehicle.

For *SSU72* knockdown, A549 cells were transfected with control siRNA (RiboBio) or an *SSU72*-specific siRNA (5ʹ-GACTCACGTGAAGCTTCCA-3ʹ) by using RNAiMAX transfection reagent (Thermo Fisher Scientific; cat. no. 13778075) according to the manufacturer’s instructions and were then treated for 48 h with the A/New Caledonia/20/1999(H1N1) virus, the A/chicken/Jilin/9/2004(H5N1) virus, or an equal volume of AF.

### Expression of viral protein fragments

HEK293T cells were transfected for 48 h with plasmids containing 10 protein-coding fragments (*PB1, PB2, PA, NP, HA, NA, M1, M2, NS1*, and *NS2*) from the H5N1 virus or A/New Caledonia/20/1999(H1N1) virus.

### CRISPRi cell lines

Monoclonal cell lines expressing dCas9 and control gRNA or a gRNA targeting *GLS*-TRT or *IL23A*-TRT were seeded into 12-well plates (for RNA extraction and protein expression analysis) or 96-well plates (for cell viability assays) at a density of 1 × 10^4^ cells per well. After 24 h, the cells were challenged with the A/New Caledonia/20/1999(H1N1) virus or A/chicken/Jilin/9/2004(H5N1) virus at an MOI of 4 or treated with an equal volume of vehicle. RNA and proteins were extracted from the 12-well plates 3, 6, 12, 18, and 24 h after infection.

### Cell viability assay

Cell viability was determined at 24 h or 48 h after infection using an [(3-(4,5-dimethylthiazol-2-yl)-5-(3-carboxymethoxyphenyl)-2-(4-sulfophenyl)-2H-tetrazolium)] (MTS) assay (Promega Biosciences Inc., San Luis Obispo, CA, USA; cat. no. G3582).

### Fluorescence in situ hybridization

In situ hybridization of the *GLS*-TRT region (440 bp) and *IL23A*-TRT region (925 bp) was performed using a *GLS*-TRT probe (5′-AAGCGAATGCTATTCCCACG-3′) and an *IL23A*-TRT probe (5′-GCTGATTGTTGGCAAAGCC-3′), respectively. Each probe was labeled with Cy3 at the 5′ end.

A549 cells grown on coverslips were treated with the A/New Caledonia/20/1999(H1N1) virus, the A/chicken/Jilin/9/2004(H5N1) virus, or an equal volume of vehicle for 24 h; washed twice with PBS; and fixed with 4% (v/v) paraformaldehyde for 20 min. After fixation, the cells were permeabilized with proteinase K in PK buffer [0.5 M EDTA, 1 M Tris-HCl (pH 7.4–7.5), and 20 mg/mL proteinase K] for 10 min and treated with 0.1 M triethanolamine for 10 min. After rinsing in PBS buffer, the cells were prehybridized in hybridization buffer (50% formamide, 5× SSC, 5× Denhardt’s solution, 250 µg/mL yeast RNA, and 500 µg/mL salmon sperm DNA) at RT for 1 h in a hybridization box. The prehybridization buffer was replaced with hybridization solution (containing 1 nM RNA probe), and the cells were incubated at 55 °C overnight. The cells were then washed three times with 0.2× SSC for 20 min at 40 °C, once with 0.2× SSC for 5 min at RT, and twice with Buffer B1 [0.1 M Tris-HCl (pH 7.4–7.5) and 150 mM NaCl] for 5 min at room temperature. Finally, the cells were washed twice with PBS and mounted with ProLong Gold Antifade Reagent (Thermo Fisher Scientific; cat. no. P10144) and DAPI (Thermo Fisher Scientific; cat. no. D3571). Image acquisition and analysis were performed using a Zeiss LSM780 confocal microscope system (Thornwood, NY, USA). Fluorescence was semiquantitatively assessed on the basis of the mean fluorescence intensity (MFI) of each cell.

### Immunofluorescence studies

HEK293T cells were grown on glass chamber slides; transfected with the SSU72-Myc, SSU72-Myc, and GFP, or SSU72-Myc and NS1-GFP expression plasmids (0.2 μg/well in 48-well plates); and fixed after 48 h with 4% (v/v) paraformaldehyde for 20 min. After fixation, the cells were washed with PBS buffer and were then permeabilized with 0.1% Triton X-100 for 10 min. After the cells were washed with PBS buffer, they were blocked with blocking buffer (containing 5% bovine serum albumin) at room temperature for 1 h. SSU72-Myc was detected by staining the cells with an anti-Myc mAb (1:2000; Cell Signaling Technology, cat. no. 2276) and an Alexa Fluor 568 goat anti-mouse (IgG) secondary antibody (1:1000; Abcam Inc., Cambridge, MA, USA; cat. no. ab175473). Nuclei in all cells were labeled with Hoechst 33258 (1 ng/mL). Image acquisition and analysis were performed using a Zeiss LSM780 microscope.

### Lentivirus production

HEK293T cells were cultured in 15-cm plates to 70–80% confluence (approximately 1.5 × 10^7^ cells). Then, they were cotransfected with 15 μg of the lentiviral plasmid, 15 μg of the psPAX2 plasmid (Addgene; cat. no. 12260), and 10 μg of pMD2.G plasmid (Addgene; cat. no. 12259) in the presence of a DNA transfection reagent (NEOFECT, Beijing, China). After 18 h, the medium was replaced with 25 mL of fresh complete medium. The supernatant was harvested at 48 h and 72 h, centrifuged at 1000 rpm and 4 °C for 10 min to remove cells, and filtered through a 0.45-μm filter to remove debris. Finally, the supernatant was ultracentrifuged at 120,000 × *g* for 2 h at 4 °C, the supernatant from this step was removed, and the precipitate was dissolved in PBS and stored at −80 °C.

### Generation of *SSU72* transgenic mice

SSU72-overexpressing mice were generated by Cyagen Biosciences (Guangzhou, China) and were then bred at the Institute of Basic Medical Sciences, Peking Union Medical College. All mice were housed in an SPF facility, and animal experiments were conducted at the Institute of Military Veterinary Medicine, Academy of Military Medical Sciences, in accordance with governmental and institutional guidelines.

In brief, C57BL/6J mouse embryos were injected with the pRP:ExSi-EF-1α-*SSU72* vector containing a fragment of the mouse *SSU72* gene. Pups were screened by PCR by using primers targeting *SSU72* (forward: 5ʹ-GCTTTTGGAGTACGTCGTCTTTAGGT-3ʹ; reverse: 5ʹ-CCATGCTCCGATTCTGGTTACTCG-3ʹ) and Western blotting with an antibody to confirm the insertion of the *SSU72* fragment and SSU72 overexpression in the lungs.

### Viral infection in *SSU72* transgenic mice

Lung injury was induced *via* intratracheal instillation of the A/chicken/Jilin/9/2004 (H5N1)virus (10^6^ TCID_50_) as previously described [36]. Control mice were treated with an equal volume of AF. Mice were killed 3 days after infection, and RNA was extracted from lung tissue for RNA-seq and RT-qPCR to detect viral replication. The lung viral titer was determined as previously described [33]. In addition, lung injury was assessed by pathological examination. Survival data were obtained and plotted on Kaplan–Meier survival curves.

### Assessment of lung injury

The wet lung weight was determined to assess pulmonary edema, and the lungs were then dried in an oven at 65 °C for 24 h to determine the dry weight.

For assessment of inflammatory injury, the lungs were removed from the thoracic cavity and fixed in a glass vial with 50 mL of formalin for at least 72 h. The fixed tissues were then embedded in paraffin, coronally sectioned, and mounted on glass slides by using standard techniques. The sections were stained with hematoxylin and eosin and examined by three independent pathologists who were blinded to the treatments and genotypes. Inflammatory cells were counted in 100 microscopic fields per slide, and lung injury was scored [37] based on the alveolar wall thickness, presence of hyaline membranes, and filling of the airspaces with proteinaceous debris. The score ranged from 0 to 1, where X indicated severe injury and Y indicated normal tissue.

### Western blot analysis

Cells or tissues were homogenized in ice-cold RIPA lysis buffer [50 mM Tris-HCl (pH 7.5), 150 mM NaCl, 1.0% Triton X-100, 20 mM EDTA, 1 mM Na_3_VO_4_, 1 mM NaF, and protease inhibitors], and proteins were loaded onto a gel for electrophoretic separation and transferred onto nitrocellulose membranes. The membranes were sequentially incubated with primary antibodies (described below) and horseradish peroxidase-conjugated secondary antibodies (MultiSciences Lianke Biotech Co., Hangzhou, China). Antibody binding was visualized using a Kodak detection system and analyzed using Quantity One software. Primary antibodies specific for the following proteins/peptides were used: SSU72 (1:1000; Cell Signaling Technology, cat. no. 12816) and STAT1 (1:500; Cell Signaling Technology, cat. no. 9172), STAT2 (1:500; Bethyl Laboratories, Inc., Montgomery, Texas, USA; cat. no. A303-512A-M), Flag tag (rabbit, 1:5000; MultiSciences; cat. no. LK-ab002-100), DDDDK tag (mouse, 1:5000; MBL Inc., Ottawa, ON, Canada; cat. no. M185-3 L), β-actin (1:10000; Sigma Aldrich, Saint Louis, MO, USA; cat. no. A5441), and CRISPR/Cas9 (polyclonal; Diagenode Inc., Denville, NJ, USA; cat. no. C15310258).

### Quantitative reverse transcription PCR (RT-qPCR) analysis

cDNA was synthesized from 1.5 μg of total RNA by using a High-Capacity cDNA Reverse Transcription Kit (Thermo Fisher Scientific; cat. no. 4368814), and RT–qPCR was performed using a LightCycler 480 SYBR Green I Master Kit (Roche; cat. no. 04707516001) on a LightCycler 480 PCR system. RT–qPCR products were confirmed by sequencing. Expression levels in mice were normalized to β-actin, and expression levels in human samples were normalized to glyceraldehyde 3-phosphate dehydrogenase. The primers used for RT-qPCR are listed in Table S1.

### CRISPRi

The target sequence was cloned into the lentiGuide-Puro backbone (Addgene; cat. no. 52963), and two oligonucleotides were generated by BsmBI digestion. Targets were defined using http://crispr.mit.edu to design the gRNA sequence. The KRAB domain from pHR-SFFV-dCas9-BFP-KRAB (Addgene; cat. no. 46911) was cloned into the *BamHI* and *BsrGI* sites in the lenti-dCAS-VP64_Blast (SAM; Addgene; cat. no. 6142) backbone, which features VP64 domain deletion. The following primers were used: *GLS*-TRT gRNA forward, 5ʹ-CACCGAGGACGTAGAACAACAGCGC-3ʹ and reverse, 5ʹ-AAACTCCTGCATCTTGTTGTCGCGC-3ʹ; and *IL23A*-TRT gRNA forward, 5ʹ-CACCGCCCCTGGTGTATAGAATAAC-3ʹ and reverse, 5ʹ-AAACGTTATTCTATACACCAGGGGC-3ʹ. The lentiGuide-sgRNA-Puro and lenti-dCAS-KRAB vectors were packaged into lentiviruses. A549 cells were infected with the lenti-dCAS-KRAB lentiviruses, and monoclonal cell lines were screened for transductants showing stable dCAS9 protein expression. Stably transduced dCas9-expressing cells were infected with lentivirus expressing control gRNA, *GLS*-TRT gRNA, or *IL23A*-TRT gRNA for 48 h and were then incubated with puromycin (2 μg/mL) to screen for monoclonal cell lines.

### Chromatin immunoprecipitation

Chromatin was sonicated using a Bioruptor Plus Sonicator with 10 high-power pulse cycles (15 s on and 15 s off) according to the protocols for the EZ-Magna ChIP A Kit (Millipore, Burlington, MA, USA; cat. no. 17-408). Fragmented chromatin was diluted with ChIP dilution buffer [0.01% SDS, 1.1% Triton X-100, 1.2 mM EDTA, and 16.7 mM Tris-HCl (pH 8.1)] and incubated with 2 μL of a Cas9-ChIP-grade antibody (Diagenode; cat. no. C15310258) overnight at 4 °C on a rotator. Next, 20 μL of protein A/G magnetic beads (Millipore; cat. no. 16-663) was added, and the samples were incubated with rotation for 2 h at 4 °C. The beads were then washed twice with each of the following buffers: low-salt immune complex wash buffer [0.1% SDS, 1% Triton X-100, 2 mM EDTA, 20 mM Tris-HCl (pH 8.1), and 150 mM NaCl], LiCl wash buffer [0.25 M LiCl, 1% NP-40, 1% deoxycholate, 1 mM EDTA, and 10 mM Tris-HCl (pH 8.1)], and TE (10 mM Tris-HCl and 1 mM EDTA, pH 8.0). Chromatin was eluted by incubation with elution buffer (0.2% SDS and 0.1 M NaHCO_3_ supplemented with fresh 5 mM DTT) at 65 °C for 120 min. After reversal of crosslinking, the samples were digested with proteinase K and RNase, and DNA was extracted by ethanol precipitation.

### Nuclear protein extraction and immunoprecipitation

HEK293T cells were transfected with a plasmid expressing Flag-tagged NS1 from the H5N1 virus or with control plasmid and cultured in 10-cm dishes. After 48 h, nuclear proteins were extracted using a Nuclear and Cytoplasmic Protein Extraction Kit (Beyotime Biotechnology, Jiangsu, China; cat. no. P0028).

Nuclear proteins were incubated with an anti-Flag or isotype control antibody (Beyotime Biotechnology, China) at 4 °C overnight on a rotator. Next, 20 μL of protein A-agarose beads (Santa Cruz, CA, USA; cat. no. sc2001) was added, and the mixture was incubated for an additional 2 h at 4 °C. The beads were then washed five times with TBS buffer [50 mM Tris-Cl (pH 7.5) and 150 mM NaCl] and boiled for 5 min in 60 μL of 2× loading buffer. Protein samples were loaded onto a 10% SDS–polyacrylamide gel for electrophoretic separation, and proteins were then transferred onto a nitrocellulose filter membrane. The membrane was blocked in 5% (m/v) nonfat milk in TBS-Tris buffer and sequentially incubated with primary antibodies (see the antibodies used for Western blot analysis described above) overnight and horseradish peroxidase-conjugated secondary antibodies for 1 h. Secondary antibody binding was detected using a film exposure detection system (Kodak, Rochester, NY, USA). Detection of a second primary antibody was performed by stripping the nitrocellulose membrane with stripping buffer (1% SDS and 25 mM glycine, pH 2.0) and then incubating it with another primary antibody.

### Statistical analysis

SPSS 16.0 for Windows (IBM, Chicago, USA) was used for statistical analyses. We used the one-sample Kolmogorov–Smirnov test to test for normality. ANOVA was used to determine intergroup differences in normally distributed data, the Mann–Whitney *U* test was used to determine intergroup differences in skewed data, and the chi-squared test was used to determine differences in categorical data. Pearson’s or Spearman’s rank linear correlation analysis was used to analyze the relationships between cell viability and TRT, and a least squares second-order polynomial was used to fit nonlinear relationships between the gene count and cell viability or viral replication. The Benjamini–Hochberg correction was used to correct for multiple testing in R software. A two-tailed *P* value of <0.05 was considered statistically significant.

## Results

### TRT induced by AIV infection is associated with cell lethality

**Fig. 1:**
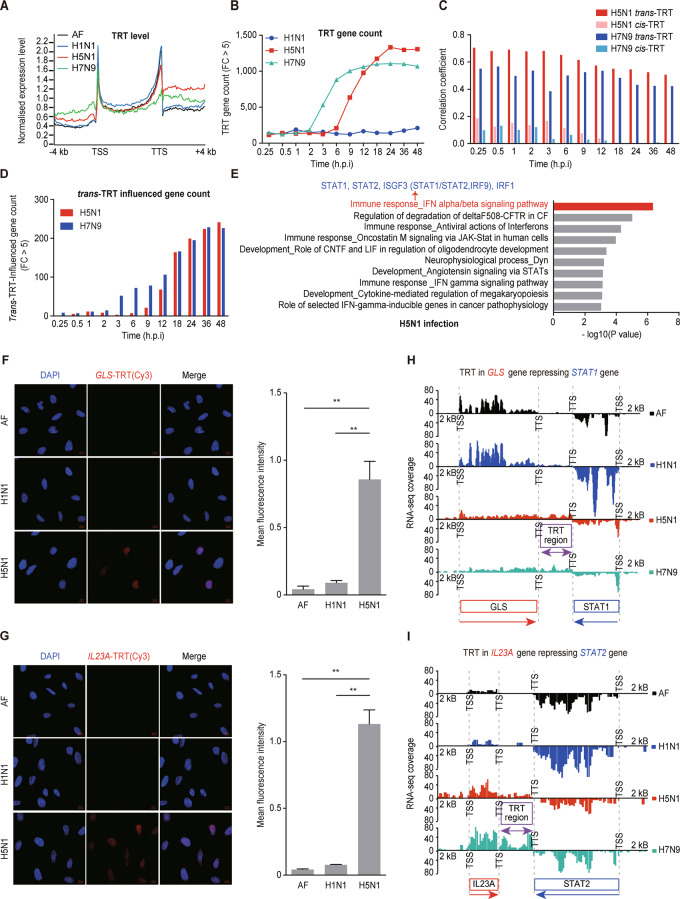
TRT enhanced by avian influenza A virus infection represses genes on the complementary DNA strand. **A** Gene profile of the averaged normalized expression levels of 5,052 genes in A549 cells at 24 h after H1N1/H5N1/H7N9 influenza virus infection or AF treatment. The gene bodies between the TSSs and TTSs were equally sized and scaled to 60 bins, and the gene flanking regions 4 kb upstream of the TSSs and 4 kb downstream of the TTSs were divided into 100-bp windows. **B** Numbers of TRT genes (FC in the expression level of the TRT region (H5N1/AF) > 5) at different times after H1N1/H5N1/H7N9 infection of A549 cells. **C** Spearman rank linear correlation coefficient between the upregulated expression levels of the TRT region and the downregulated expression levels of TRT-influenced genes following the *trans*-TRT and *cis*-TRT patterns, respectively, at different times after H5N1/H7N9 infection in A549 cells. **D** Numbers of *trans*-TRT-influenced genes (FC in the expression level of the *trans*-TRT gene (H5N1/AF) > 5 and FC in the expression level of the *trans*-TRT-influenced gene (H1N1/H5N1) > 1.5) at different times after H5N1/H7N9 infection in A549 cells. **E** Functional pathway enrichment analysis of *trans*-TRT-influenced genes in H5N1-infected A549 cells (two-tailed *P* < 0.05, Benjamini–Hochberg adjusted *P* < 0.05). Detection of **F** *GLS*-TRT or **G** *IL23A*-TRT in A549 cells by using fluorescence in situ hybridization (FISH). A549 cells were treated with AF/H1N1/H5N1 for 24 h [DAPI nuclear staining (*blue*) and FISH signals obtained using a Cy3-conjugated DNA probe (*red*)]. The fluorescence intensity was semiquantitatively assessed using the mean fluorescence intensity (MFI) of each cell. The data are shown as the means ± SEMs. *P < 0.05, **P < 0.01. RNA-seq coverage levels of **H** the *GLS* gene, *trans*-TRT region of *GLS*, and *STAT1* gene, and **I** the *IL23A* gene, *trans*-TRT region of *IL23A*, and *STAT2* gene 12 h after AF/H1N1/H5N1/H7N9 treatment of A549 cells. The gene bodies and intergenic regions, as well as the gene flanking regions 2 kb upstream of the TSSs, were divided into 50-bp windows. Only exon regions are shown in this graph. RNA-seq datasets were established in duplicate

To explore the transcriptional changes induced by influenza A virus infection, we performed RNA-seq on human A549 lung carcinoma cells infected with H1N1, H5N1, or H7N9 virus. TRT was higher in cells infected with AIVs (H5N1 and H7N9) than in control cells, but no significant difference was detected between H1N1-infected cells and control cells (Figs. 1A and S1A–C). The number of genes with TRT was higher in H5N1- and H7N9-infected cells than in H1N1-infected cells (Fig. 1B).

TRT was negatively correlated with cell viability and positively correlated with H5N1 and H7N9 virus replication (Fig. S1D–G). In experiments involving PBMCs, human cancer cell lines (A549, H1650, and HeLa), kidney cell lines (HEK293 and HEK293T), a human acute monocytic leukemia cell line (THP-1), a human T lymphocyte cell line (Jurkat), and a primary fibroblast line (HPF-a), infection with H5N1 was associated with a higher TRT level than infection with H1N1, except in HeLa cells, in which AIV infection was minimally detected (Fig. S1H, I). These findings suggest that TRT may be negatively correlated with the viability of influenza A virus-infected cells (Fig. S1J).

### TRT represses host antiviral gene expression

To determine the potential roles of TRT in AIV infection, we performed correlation analysis between TRT levels and downregulated DEGs in infected A549 cells. Because TRT spans the promoter and coding regions of *cis-* and *trans-*genes, it might affect the expression of downstream genes on the same DNA strand (*cis*-TRT; Fig. S2A) or on the complementary DNA strand (*trans*-TRT; Fig. S2B). To determine which genes were the most strongly influenced by AIV infection, we calculated the correlations between TRT levels and *cis*- and *trans*-gene expression levels (Figs. 1C and S2C). *Trans-*gene expression was more strongly correlated with cell infection than *cis*-gene expression, indicating that TRT might specifically regulate the expression of genes on the complementary strand in infected cells.

The number of *trans*-TRT-influenced genes in H5N1- and H7N9-infected cells was substantially increased at 12 h after infection (Fig. 1D). Furthermore, the number of *trans*-TRT-influenced genes was positively correlated with viral replication and negatively correlated with cell viability in H5N1-infected cells (Fig. S2D, E), suggesting that TRT is correlated with viral lethality.

Functional analysis of genes influenced by *trans*-TRT showed that among the viral strains, H5N1 infection resulted in the greatest dysregulation of genes involved in innate immunity (Fig. 1E) and that H7N9 infection also affected immune gene expression (Fig. S2F). These results suggested that *trans*-TRT-related genes might be responsible for the high lethality associated with H5N1 virus infection and contribute to the disruption of host innate immunity.

*Trans*-TRT during H5N1 infection resulted in the greatest decreases in the expression of *STAT1* and *STAT2*, which are associated with the IFN-α/β signaling pathway. We performed fluorescence in situ hybridization by using probes targeting the TRT regions downstream of the TTSs in *GLS* and *IL23A* (their sequences are shown in Table S1), which correspond to the *trans*-TRT positions in *STAT1* and *STAT2*, and then performed RT–qPCR with primers targeting the TRT regions to confirm that TRT caused transcription to continue past the TTS in *GLS* and *IL23A* (Figs. 1F, G and S3A–C). Investigation of the expression levels of *STAT1* and *STAT2* in H1N1- and H5N1-infected cells showed that their expression was repressed by H5N1 infection but elevated by H1N1 infection (Figs. 1H-I and S4A–D). These data suggested that TRT influenced the expression of innate immune genes, especially *STAT1* and *STAT2*, on the complementary strand through *trans-*TRT.

### Inhibition of TRT promotes STAT1/STAT2 expression

To confirm the function of TRT in repressing *STAT1* and *STAT2* expression, we used CRISPR interference (CRISPRi) [38–40] to abolish TRT in *GLS* and *IL23A* in H5N1-infected A549 cells and then investigated *STAT1* and *STAT2* expression. CRISPRi was used to express a catalytically inactive CRISPR-associated endonuclease Cas9 (dCas9), which was targeted by guide RNAs to sequences between the TTSs and TRT termination sites of the *GLS* and *IL23A* genes (Fig. S5A, B). We confirmed the correct targeting of inhibitory complexes by using ChIP-PCR with an anti-Cas9 antibody and PCR primers targeting the TRT regions of *GLS* and *IL23A* (Fig. S5C, D; the sequences are listed in Table S1).

**Fig. 2:**
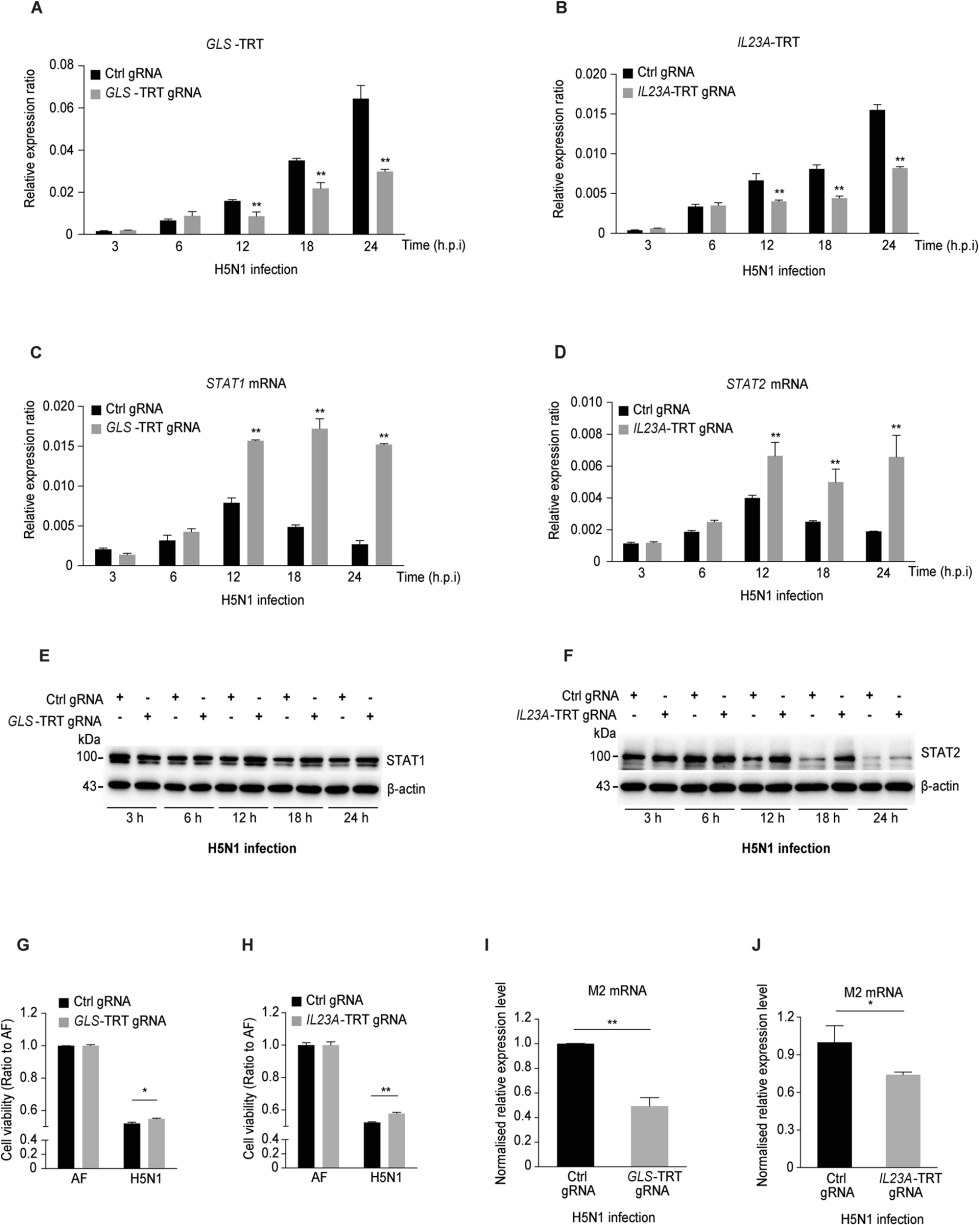
TRT inhibition by CRISPR interference enhances STAT1/STAT2 expression and cell viability. RT-qPCR analysis of the **A** *GLS*-TRT gRNA and **B** *IL23A*-TRT gRNA groups at different times after infection with H5N1 (MOI = 4). RT-qPCR analysis of **C** *STAT1* mRNA expression in the Ctrl gRNA and *GLS*-TRT gRNA groups and **D** *STAT2* mRNA expression in the Ctrl gRNA and *IL23A*-TRT gRNA groups at different times after infection with H5N1 (MOI = 4). Western blot analysis of **E** STAT1 protein expression in the Ctrl gRNA and *GLS*-TRT gRNA groups and **F** STAT2 protein expression in the Ctrl gRNA and *IL23A*-TRT gRNA groups at different times after infection with H5N1 (MOI = 4). β-Actin expression served as the reference control. MTS cell viability assay in the **G** *GLS*-TRT gRNA and **H** *IL23A*-TRT gRNA groups at 48 h after treatment with AF or infection with H5N1 (MOI = 4). RT-qPCR analysis of viral *M2* expression levels in the **I** *GLS*-TRT gRNA and **J** *IL23A*-TRT gRNA groups at 24 h after infection with H5N1 (MOI = 4). The expression levels in **I** and **J** are normalized to the Ctrl gRNA group. Each experiment was repeated at least three times. The data are shown as the means ± SEMs. *P < 0.05, **P < 0.01

A549 cells with CRISPRi targeting the *GLS* and *IL23A* TRT regions exhibited less TRT than control cells with untargeted CRISPRi (Fig. 2A, B). In addition, the STAT1 and STAT2 mRNA (Fig. 2C, D) and protein (Fig. 2E, F) levels were higher in CRISPRi cells than in control cells. After H5N1 infection, CRISPRi cells showed higher viability and less viral replication than control cells; however, after H1N1 infection, the CRISPRi and control cells showed similar viability and viral replication ability (Figs. 2G–J and S5E–L). These data showed that TRT of *GLS* and *IL23A* repressed the transcription of the antiviral genes *STAT1* and *STAT2* on the complementary DNA strand.

### NS1 binds to SSU72 to enhance TRT

**Fig. 3:**
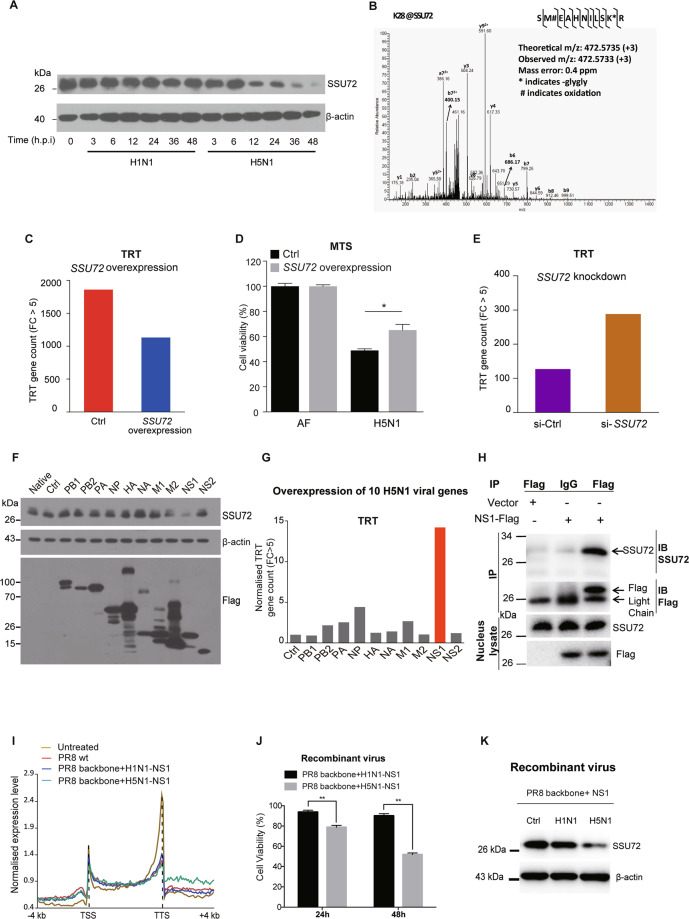
NS1 binds to SSU72 and enhances TRT. **A** Western blot analysis of SSU72 expression in H1N1/H5N1-infected A549 cells. β-Actin was used as the reference control. **B** Ubiquitination of SSU72 was identified by immunoaffinity enrichment coupled with LC–MS/MS. **C** Numbers of *trans*-TRT genes (FC > 5) in the *SSU72* overexpression and control groups at 48 h after H5N1 infection of A549 cells. **D** MTS assay of cell viability in the *SSU72* overexpression and control groups at 48 h after AF treatment or H5N1 infection. **E** Numbers of *trans*-TRT genes (FC > 5) in the *SSU72* knockdown and control groups at 48 h after AF treatment of A549 cells. **F**, **G** Overexpression of the H5N1 viral gene segments for 48 h in HEK293T cells. **F** Western blot analysis of SSU72 expression (with β-actin as the reference control). **G** Normalized numbers of *trans*-TRT genes (FC > 5). **H** Coimmunoprecipitation (co-IP) of SSU72 and the H5N1 NS1 protein in the cell nucleus; the input levels are shown. **I** Gene profile analysis of the averaged normalized expression levels in A549 cells at 48 h after treatment with or without PR8 wt virus, PR8+H1N1-NS1, and PR8+H5N1-NS1 recombinant viruses. The gene bodies between the TSSs and TTSs were equally sized to 60 bins, and the gene flanking regions 4 kb upstream of the TSSs and 4 kb downstream of the TTSs were divided into 100-bp windows. **J** MTS cell viability assay in the recombinant virus (PR8+H1N1-NS1 or PR8+H5N1-NS1) groups at 24 h or 48 h after viral infection. **K** Western blot analysis of SSU72 at 48 h after recombinant virus infection. β-Actin was used as the reference control. The data are shown as the means ± SEMs. *P < 0.05 and **P < 0.01. Each experiment except for RNA-seq analysis of recombinant virus-infected cells was repeated at least three times

While investigating the mechanism underlying TRT, we found that SSU72 protein expression was markedly decreased after H5N1 infection but not after H1N1 infection (Fig. 3A). Interestingly, TRT occurred before SSU72 protein degradation. Our posttranslational modification analysis results indicated that SSU72 was associated with a ubiquitin complex during H5N1 infection (Fig. 3B). After H5N1 infection, SSU72-overexpressing A549 cells showed less TRT (Fig. 3C), higher viability (Fig. 3D), and less viral replication (Fig. S6A, B) than control A549 cells. In addition, SSU72-overexpressing A549 cells had lower *GLS* and *IL23A* TRT levels and higher *STAT1*, *STAT2*, and IFN-β expression levels than control A549 cells (Fig. S6C–G).

Next, we used small interfering RNAs to knock down *SSU72* expression in A549 cells (Fig. S6H). TRT occurred in more genes in cells with *SSU72* knockdown than in control cells (Fig. 3E). After H1N1 or H5N1 infection, A549 cells with *SSU72* knockdown showed lower viability (Fig. S6I, J), higher *GLS* and *IL23A* TRT levels (Fig. S6K–N), lower *STAT1/2* mRNA levels (Fig. S6O–R), and less viral replication (Fig. S6S, T) than cells with normal *SSU72* expression. These results suggested that SSU72 is a crucial regulator of TRT during AIV infection.

To determine the precise mechanisms by which SSU72 regulates TRT, we overexpressed 10 H5N1 genes in HEK293T cells and performed RNA-seq to analyze TRT levels. Although exogenous expression of each viral protein resulted in different TRT levels, cells overexpressing the H5N1 NS1 protein had higher TRT levels and lower SSU72 expression levels than control cells (Fig. 3F, G). In contrast, overexpression of H1N1 proteins did not increase TRT levels (Fig. S7A, B).

Coimmunoprecipitation and confocal microscopy imaging showed that the H5N1 NS1 protein bound directly to the human SSU72 protein (Figs. 3H and S7C). To test whether this binding was unique to the H5N1 NS1 protein, the PR8+H5N1-NS1 and PR8+H1N1-NS1 were used for comparative test. The TRT levels were increased in A549 cells infected with the PR8+H5N1-NS1, suggesting that H5N1 NS1 plays a key role in inducing TRT during infection (Fig. 3I). In addition, cell viability and SSU72 expression were decreased in A549 cells infected with the PR8+H5N1-NS1 compared with those infected with the PR8 wt virus and PR8+H1N1-NS1 (Fig. 3J, K). These results suggested that NS1 binds to SSU72, leading to a reduction in its expression, which eventually induces TRT and represses *STAT1/2* mRNA expression (Fig. S7D, E).

### Dampened innate immune gene expression upon AIV infection is restored in *SSU72-*overexpressing transgenic mice

**Fig. 4:**
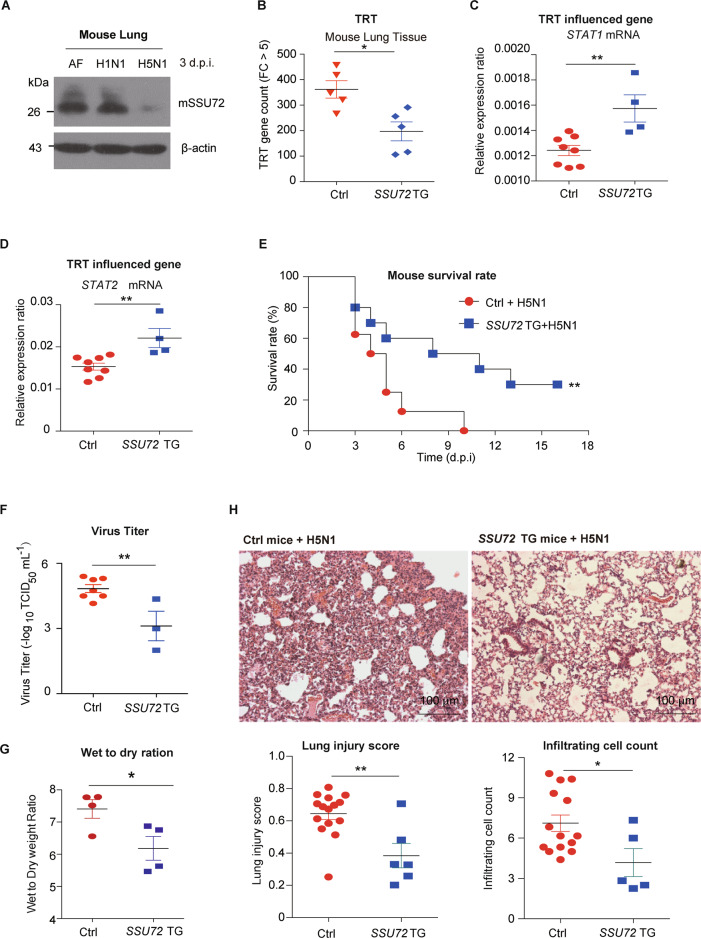
TRT is reduced and lung injury is ameliorated in *SSU72* transgenic mice infected with the lethal H5N1 virus. **A** Western blot analysis of mouse SSU72 expression in mouse lung tissues at 3 days after treatment with AF/H1N1/H5N1. β-Actin expression served as an internal control. **B** Numbers of TRT genes (expression of the TRT region upregulated by more than 5 compared with the AF-treated condition) in lung tissues from control (*n* = 5) and *SSU72* transgenic mice (*n* = 5) at 3 days after intratracheal infection with H5N1 (10^6^ TCID_50_). The relative mRNA expression ratios of **C** mouse *STAT1* and **D** *STAT2* in lung tissues from control (*n* = 8) and *SSU72* transgenic mice (*n* = 4) at 3 days after intratracheal infection with H5N1 virus (10^6^ TCID_50_). Mouse β-actin expression served as the reference control. **E** Kaplan–Meier survival curves for control (*n* = 8) and *SSU72* transgenic mice (*n* = 10) after intratracheal infection with H5N1 (10^6^ TCID_50_). **F–H** Control and *SSU72* transgenic mice were infected with AF or H5N1 (10^6^ TCID_50_) *via* intratracheal instillation. **F** Viral titers in the lungs were assessed 4 days after infection with H5N1 in control (n = 7) and *SSU72* transgenic mice (n = 3). **G** Wet-to-dry weight ratios of the lungs of control (*n* = 4) and *SSU72* transgenic mice (*n* = 4) at 3 days after infection with H5N1. **H** Representative images of lung pathology in control and *SSU72* transgenic mice at 3 days after H5N1 infection. The lung injury scores (means ± SEMs) and numbers of infiltrating cells per microscopic field (means ± SEMs) are shown in the bar graphs. *N* = 100 fields for control (*n* = 15) and *SSU72* transgenic (*n* = 6) mice. Bar = 100 μm. *P < 0.05 and **P < 0.01. Each experiment except for RNA-seq analysis of lungs from mice with or without H5N1 infection was repeated at least three times

In the in vivo studies, SSU72 expression levels in lung tissues were lower in mice infected with H5N1 than in those infected with H1N1 (Fig. 4A). To further elucidate the role of SSU72 in influenza A virus infection, we generated transgenic *SSU72* mice with *SSU72* overexpression in the lungs and infected them with H5N1 (Fig. S8A). RNA-seq analysis of mouse lung tissues showed that the TRT gene count was lower in *SSU72* transgenic mice than in control mice (Fig. 4B). In addition, *SSU72* transgenic mice showed higher lung expression levels of *STAT1* and *STAT2* (Fig. 4C, D), less severe lung injury and edema, lower viral titers, and higher survival rates than control C57BL/6J mice (Fig. 4E–H).

These results suggested that overexpression of SSU72 can inhibit TRT in mouse lung tissue during viral infection; this might be a potential strategy for preventing future AIV pandemics.

### TRT is increased in blood samples from AIV-infected patients

**Fig. 5:**
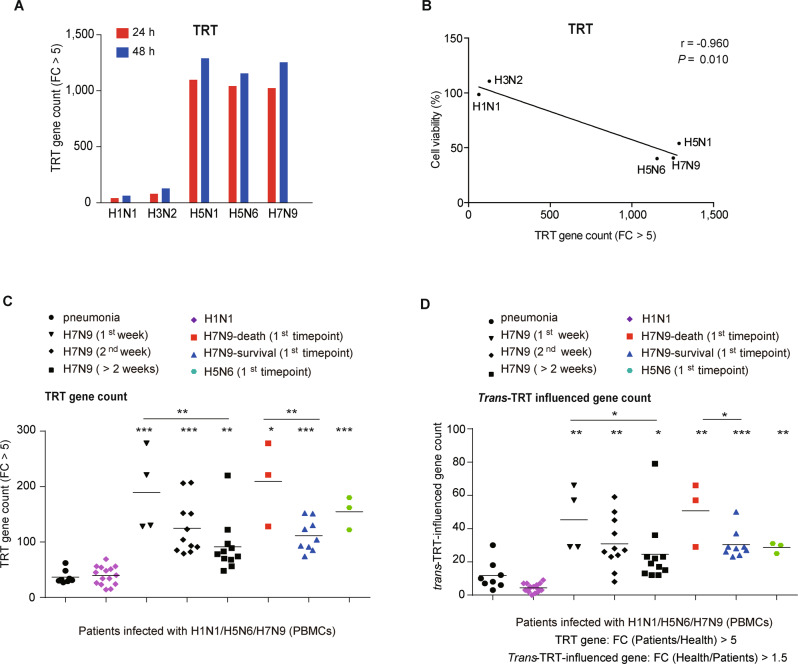
TRT is enhanced in PBMCs from patients infected with influenza virus. **A** Numbers of TRT genes (those with TRT region expression upregulated by more than 5-fold after AIV infection compared with AF treatment) in A549 cells at 24 h and 48 h after H1N1/H3N2/H5N1/H5N6/H7N9 infection. **B** Correlation analysis between the number of TRT genes (those with TRT region expression upregulated by more than fivefold after AIV infection compared with AF treatment) and cell viability in A549 cells at 48 h after H1N1/H3N2/H5N1/H5N6/H7N9 infection. A549 cell viability was determined using an MTS assay. The Pearson correlation coefficients (r) and *P* values are provided in the graph. **C** Numbers of TRT genes (those with TRT region expression upregulated by more than fivefold in the patient group compared with the healthy control group) and **D** numbers of *trans*-TRT-influenced genes (those with TRT region expression upregulated with a FC greater than 5 in the patient group compared with the healthy control group or downregulated by more than 1.5-fold in the patient group compared with the healthy control group) in human PBMCs. The horizontal lines indicate the mean values in each group. ANOVA was used for comparisons among multiple groups. *P < 0.05, **P < 0.01, and ***P < 0.001. All experiments except for those involving human blood samples were performed at least in triplicate

To determine whether TRT is associated with influenza virus lethality, we infected A549 cells with highly virulent or less virulent viruses and then analyzed TRT levels. TRT levels increased strongly after infection with the lethal avian viruses H5N1, H5N6, and H7N9 and moderately after infection with the less virulent viruses H1N1 and H3N2 (Fig. 5A). TRT was also negatively correlated with cell viability and positively correlated with viral replication (Figs. 5B and S8B). These results suggested that TRT is associated with high viral lethality.

To confirm our laboratory results in the clinic, we obtained 29 blood samples from 12 H7N9-infected patients, 3 blood samples from 3 H5N6-infected patients, 15 blood samples from 15 H1N1-infected patients, 8 blood samples from 8 patients with pneumonia (without influenza virus infection), and 10 blood samples from healthy individuals (see Table S2 for the patient characteristics). RNA-seq of the isolated PBMCs revealed that the *TRT* gene count was higher in PBMCs from patients infected with H7N9 and H5N6 than in the other PBMC samples analyzed (Fig. 5C). In addition, the *trans*-TRT genes were more repressed in PBMCs from the H7N9- and H5N6-infected patients than in those from the other patients (Fig. 5D). Notably, the TRT levels and the number of suppressed *trans*-TRT genes were higher in H7N9-infected patients who died than in those who survived. Our results suggested that TRT might be a potential biomarker for the outcomes of patients with AIV infection.

## Discussion

In this study, we showed that TRT was enhanced by AIV infection in cultured human cells, mouse lung tissues, and patient-derived PBMCs. TRT inhibited the expression of innate immune genes on the complementary DNA strand, a phenomenon that we called *trans*-TRT. TRT also inhibited the expression of immune response-related genes such as *STAT1* and *STAT2* in cell lines and mouse lung tissues. This finding does not contradict a previous discovery that NS1 can repress STAT1/2 through IFNα/β [14, 41–45]. Both mechanisms could coexist or exist independently in different viral strains. Further studies are required to elucidate why TRT acts mainly in *trans* and not in *cis*. Future studies should also explore other genes that are associated with the innate immune response and are influenced by *trans*-TRT.

We found that SSU72, a gene loop regulator at TTSs, plays a crucial role in TRT regulation and influences the expression of genes involved in the innate immune response both in vitro and in vivo. SSU72 binds to promoter and terminator regions to maintain gene loop formation and transcriptional directionality [23, 46]. We found that the H5N1 NS1 protein can directly bind to SSU72 and reduce its expression. This appears to be a novel mechanism by which AIVs can suppress innate immunity. Moreover, overexpression of SSU72 ameliorated lung injury and protected against suppression of antiviral gene expression in mice infected with H5N1. Further studies are necessary to elucidate the specificity of SSU72-restricted transcripts. Based on our findings, we propose that SSU72 may be a useful therapeutic target in AIV. The PBMCs of patients infected with H7N9 and H5N6 showed enhanced TRT, suggesting that TRT levels are associated with patient outcomes.

In conclusion, we showed that NS1 bound directly to SSU72 and suppressed STAT1/2 expression through *trans*-TRT, which enabled the virus to evade host antiviral immune responses, eventually leading to viral lethality. In addition to the pathways through which NS1 targets IFN by inhibiting JAK/STAT signaling, this novel mechanism constitutes an alternative pathway of viral escape from antiviral responses. Thus, we identified a novel mechanism by which TRT suppresses the innate immune system during AIV infection.

## Supplementary information

Supplementary Information

## References

[1] Peiris JS, de Jong MD, Guan Y (2007). Avian influenza virus (H5N1): a threat to human health. Clin Microbiol Rev.

[2] Chen H, Liu S, Liu J, Chai C, Mao H, Yu Z (2016). Nosocomial co-transmission of avian influenza A(H7N9) and A(H1N1)pdm09 viruses between 2 patients with hematologic disorders. Emerg Infect Dis.

[3] Li J, Yu X, Pu X, Xie L, Sun Y, Xiao H (2013). Environmental connections of novel avian-origin H7N9 influenza virus infection and virus adaptation to the human. Sci China Life Sci.

[4] Liu J, Xiao H, Wu Y, Liu D, Qi X, Shi Y (2014). H7N9: a low pathogenic avian influenza A virus infecting humans. Curr Opin Virol.

[5] Medina RA, Garcia-Sastre A (2011). Influenza A viruses: new research developments. Nat Rev Microbiol.

[6] Poovorawan Y, Pyungporn S, Prachayangprecha S, Makkoch J (2013). Global alert to avian influenza virus infection: from H5N1 to H7N9. Pathog Glob Health.

[7] Fernandez-Sesma A, Marukian S, Ebersole BJ, Kaminski D, Park MS, Yuen T (2006). Influenza virus evades innate and adaptive immunity via the NS1 protein. J Virol.

[8] Marc D (2014). Influenza virus non-structural protein NS1: interferon antagonism and beyond. J Gen Virol.

[9] Hale BG, Randall RE, Ortin J, Jackson D (2008). The multifunctional NS1 protein of influenza A viruses. J Gen Virol.

[10] Nemeroff ME, Barabino SM, Li Y, Keller W, Krug RM (1998). Influenza virus NS1 protein interacts with the cellular 30 kDa subunit of CPSF and inhibits 3’end formation of cellular pre-mRNAs. Mol Cell.

[11] Qiu Y, Nemeroff M, Krug RM (1995). The influenza virus NS1 protein binds to a specific region in human U6 snRNA and inhibits U6-U2 and U6-U4 snRNA interactions during splicing. RNA..

[12] Gack MU, Albrecht RA, Urano T, Inn KS, Huang IC, Carnero E (2009). Influenza A virus NS1 targets the ubiquitin ligase TRIM25 to evade recognition by the host viral RNA sensor RIG-I. Cell Host Microbe.

[13] Pindel A, Sadler A (2011). The role of protein kinase R in the interferon response. J Interferon Cytokine Res.

[14] Jia D, Rahbar R, Chan RW, Lee SM, Chan MC, Wang BX (2010). Influenza virus non-structural protein 1 (NS1) disrupts interferon signaling. PLoS ONE.

[15] Pauli EK, Schmolke M, Wolff T, Viemann D, Roth J, Bode JG (2008). Influenza A virus inhibits type I IFN signaling via NF-kappaB-dependent induction of SOCS-3 expression. PLoS Pathog.

[16] Fortes P, Beloso A, Ortin J (1994). Influenza virus NS1 protein inhibits pre-mRNA splicing and blocks mRNA nucleocytoplasmic transport. EMBO J.

[17] Neve J, Patel R, Wang Z, Louey A, Furger AM (2017). Cleavage and polyadenylation: ending the message expands gene regulation. RNA Biol.

[18] Dichtl B, Blank D, Ohnacker M, Friedlein A, Roeder D, Langen H (2002). A role for SSU72 in balancing RNA polymerase II transcription elongation and termination. Mol Cell.

[19] Steinmetz EJ, Brow DA (2003). Ssu72 protein mediates both poly(A)-coupled and poly(A)-independent termination of RNA polymerase II transcription. Mol Cell Biol.

[20] Zhang DW, Mosley AL, Ramisetty SR, Rodriguez-Molina JB, Washburn MP, Ansari AZ (2012). Ssu72 phosphatase-dependent erasure of phospho-Ser7 marks on the RNA polymerase II C-terminal domain is essential for viability and transcription termination. J Biol Chem.

[21] Sun ZW, Hampsey M (1996). Synthetic enhancement of a TFIIB defect by a mutation in SSU72, an essential yeast gene encoding a novel protein that affects transcription start site selection in vivo. Mol Cell Biol.

[22] Ganem C, Devaux F, Torchet C, Jacq C, Quevillon-Cheruel S, Labesse G (2003). Ssu72 is a phosphatase essential for transcription termination of snoRNAs and specific mRNAs in yeast. EMBO J.

[23] Chen Y, Zhang L, Estaras C, Choi SH (2014). Moreno LJr, Karn J, et al. A gene-specific role for the Ssu72 RNAPII CTD phosphatase in HIV-1 Tat transactivation. Genes Dev.

[24] Kuehner JN, Pearson EL, Moore C (2011). Unravelling the means to an end: RNA polymerase II transcription termination. Nat Rev Mol Cell Biol.

[25] Han Y, Lin YB, An W, Xu J, Yang HC, O'Connell K (2008). Orientation-dependent regulation of integrated HIV-1 expression by host gene transcriptional readthrough. Cell Host Microbe.

[26] Rutkowski AJ, Erhard F, L'Hernault A, Bonfert T, Schilhabel M, Crump C (2015). Widespread disruption of host transcription termination in HSV-1 infection. Nat Commun.

[27] van Luenen HG, Farris C, Jan S, Genest PA, Tripathi P, Velds A (2012). Glucosylated hydroxymethyluracil, DNA base J, prevents transcriptional readthrough in Leishmania. Cell..

[28] Grosso AR, Leite AP, Carvalho S, Matos MR, Martins FB, Vitor AC (2015). Pervasive transcription read-through promotes aberrant expression of oncogenes and RNA chimeras in renal carcinoma. Elife..

[29] Vilborg A, Sabath N, Wiesel Y, Nathans J, Levy-Adam F, Yario TA (2017). Comparative analysis reveals genomic features of stress-induced transcriptional readthrough. Proc Natl Acad Sci USA.

[30] Vilborg A, Passarelli MC, Yario TA, Tycowski KT, Steitz JA (2015). Widespread Inducible Transcription Downstream of Human Genes. Mol Cell.

[31] Zhao N, Sebastiano V, Moshkina N, Mena N, Hultquist J, Jimenez-Morales D (2018). Influenza virus infection causes global RNAPII termination defects. Nat Struct Mol Biol.

[32] Heinz S, Texari L, Hayes MGB, Urbanowski M, Chang MW, Givarkes N (2018). Transcription elongation can affect genome 3D structure. Cell..

[33] Sun Y, Li C, Shu Y, Ju X, Zou Z, Wang H (2012). Inhibition of autophagy ameliorates acute lung injury caused by avian influenza A H5N1 infection. Sci Signal.

[34] Bi Y, Xie Q, Zhang S, Li Y, Xiao H, Jin T (2015). Assessment of the internal genes of influenza A (H7N9) virus contributing to high pathogenicity in mice. J Virol.

[35] Haas J, Park EC, Seed B (1996). Codon usage limitation in the expression of HIV-1 envelope glycoprotein. Curr Biol.

[36] Kuba K, Imai Y, Rao S, Gao H, Guo F, Guan B (2005). A crucial role of angiotensin converting enzyme 2 (ACE2) in SARS coronavirus-induced lung injury. Nat Med.

[37] Matute-Bello G, Downey G, Moore BB, Groshong SD, Matthay MA, Slutsky AS (2011). An official American Thoracic Society workshop report: features and measurements of experimental acute lung injury in animals. Am J Respir Cell Mol Biol.

[38] Bikard D, Jiang W, Samai P, Hochschild A, Zhang F, Marraffini LA (2013). Programmable repression and activation of bacterial gene expression using an engineered CRISPR-Cas system. Nucleic Acids Res.

[39] Gilbert LA, Larson MH, Morsut L, Liu Z, Brar GA, Torres SE (2013). CRISPR-mediated modular RNA-guided regulation of transcription in eukaryotes. Cell..

[40] Gilbert LA, Horlbeck MA, Adamson B, Villalta JE, Chen Y, Whitehead EH (2014). Genome-scale CRISPR-mediated control of gene repression and activation. Cell..

[41] Stark GR, Darnell JE (2012). The JAK-STAT pathway at twenty. Immunity..

[42] Cheon H, Holvey-Bates EG, Schoggins JW, Forster S, Hertzog P, Imanaka N (2013). IFNbeta-dependent increases in STAT1, STAT2, and IRF9 mediate resistance to viruses and DNA damage. EMBO J.

[43] Darnell JE, Kerr IM, Stark GR (1994). Jak-STAT pathways and transcriptional activation in response to IFNs and other extracellular signaling proteins. Science..

[44] Dupuis S, Jouanguy E, Al-Hajjar S, Fieschi C, Al-Mohsen IZ, Al-Jumaah S (2003). Impaired response to interferon-alpha/beta and lethal viral disease in human STAT1 deficiency. Nat Genet.

[45] Finbloom DS, Larner AC (1995). Regulation of the Jak/STAT signalling pathway. Cell Signal.

[46] Tan-Wong SM, Zaugg JB, Camblong J, Xu Z, Zhang DW, Mischo HE (2012). Gene loops enhance transcriptional directionality. Science..

