# Supplementary material for: Avian influenza viruses suppress innate immunity by inducing trans-transcriptional readthrough via SSU72
Source: Cell Mol Immunol. 2022 Mar 24;19(6):702–14. doi: 10.1038/s41423-022-00843-8 (PMC9151799; doi:10.1038/s41423-022-00843-8)
Supplement: Supplementary file 1 — Supplementary Information [file 41423_2022_843_MOESM1_ESM.docx]

**Supplementary Information:**

**Title:** Avian influenza viruses suppress innate immunity by inducing transcriptional read-through via SSU72

It includes:

**Figure S1-S8**

**Table S1-S2**

**Supplementary Figures**

**
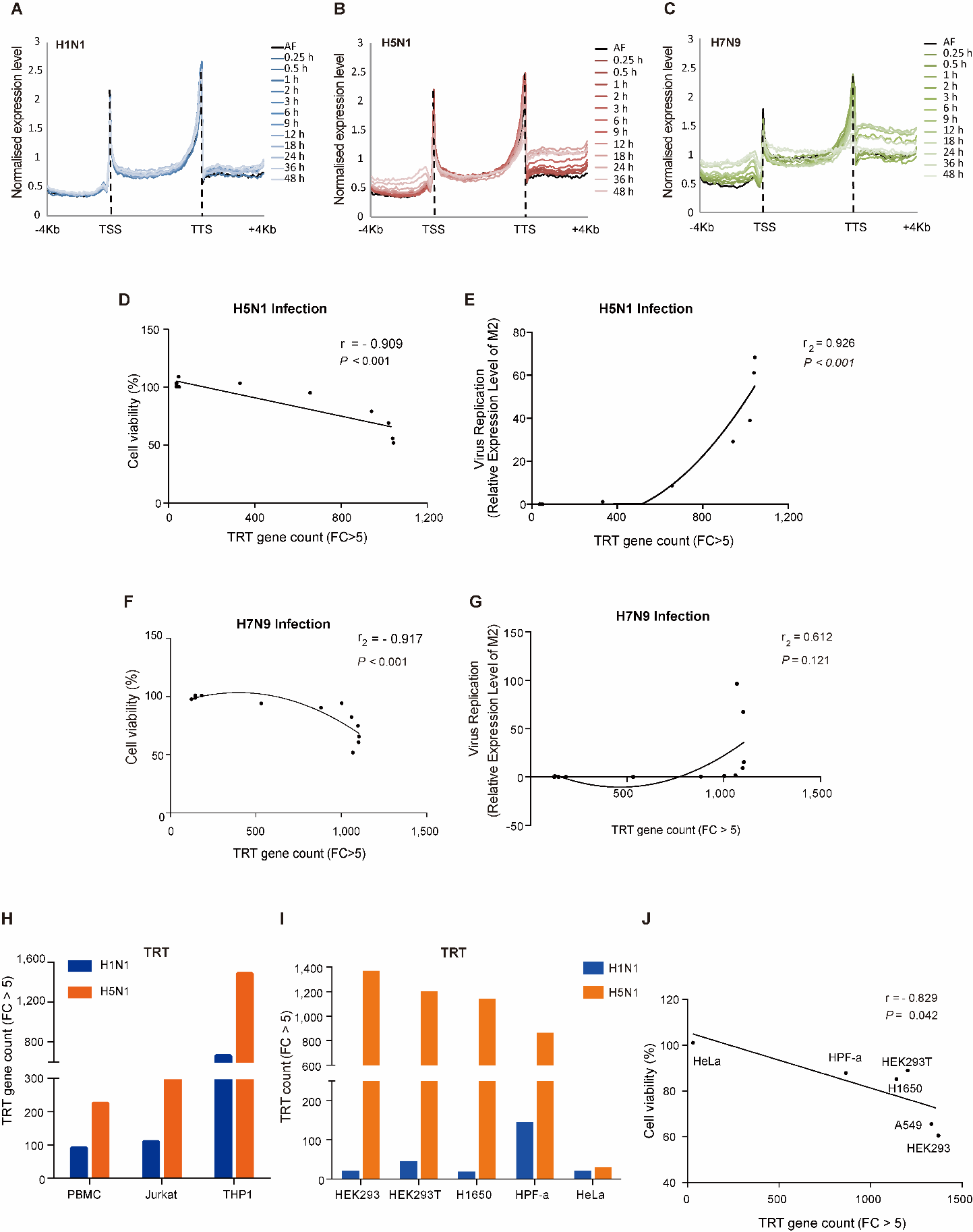
**

**Fig. S1 TRT is enhanced by avian influenza A virus infection in A549 cells and other cell lines. (A-C)** Gene profile analysis of the average normalised expression levels of 5,052 genes after AF treatment, or at 15 min, 30 min, 1 h, 2 h, 3 h, 6 h, 9 h, 12 h, 18 h, 24 h, 36 h, and 48 h after **(A)** H1N1, **(B)** H5N1, or **(C)** H7N9 influenza virus infection in A549 cells. The gene-body regions between the TSSs and TTSs were equally sized to 60 bins, and the gene-flanking regions 4 Kb upstream of TSSs and 4 Kb downstream of TTSs were spliced into 100-bp windows. **(D)** Correlation analysis between the numbers of TRT genes (TRT region expression levels upregulated greater than 5-fold compared with the AF-treated condition) and cell viability at 0 h, 15 min, 30 min, 1 h, 2 h, 3 h, 6 h, 9 h, 12 h, 18 h, 24 h, 36 h, and 48 h after H5N1 influenza virus infection in A549 cells. A549 cell viability was determined using MTS assay. Pearson correlation coefficient (r) and *P* value are provided in the graph. **(E)** Correlation analysis between the number of TRT genes (TRT region expression levels upregulated greater than 5-fold compared with the AF-treated condition) and virus replication (relative expression level of virus M2) at 0 h, 15 min, 30 min, 1 h, 2 h, 3 h, 6 h, 9 h, 12 h, 18 h, 24 h, 36 h, and 48 h after H5N1 influenza virus infection in A549 cells. The H5N1 virus *M2* gene was tested using RT-PCR to determine the virus replication level. Least squares analysis was used to fit second-order polynomial functions, and the correlation coefficient (r_2_) and *P* value are provided in the graph. **(F)** Correlation analysis between the numbers of TRT genes (TRT region expression levels upregulated greater than 5-fold compared with the AF-treated condition) and cell viability at 0 h, 15 min, 30 min, 1 h, 2 h, 3 h, 6 h, 9 h, 12 h, 18 h, 24 h, 36 h, and 48 h after H7N9 influenza virus infection in A549 cells. A549 cell viability was determined using MTS assay. Least squares analysis was used to fit second-order polynomial functions, and the correlation coefficient (r_2_) and *P* value are provided in the graph. **(G)** Correlation analysis between the number of TRT genes (TRT region expression levels upregulated greater than 5-fold compared with the AF-treated condition) and virus replication (relative expression level of virus *M2*) at 0 h, 15 min, 30 min, 1 h, 2 h, 3 h, 6 h, 9 h, 12 h, 18 h, 24 h, 36 h, and 48 h after H7N9 influenza virus infection in A549 cells. The H7N9 virus *M2* gene was tested using real-time PCR to determine the virus replication level. Least squares analysis was used to fit second-order polynomial functions, and the correlation coefficient (r_2_) and *P* value are provided in the graph. **(H-I)** Numbers of TRT genes (TRT region expression levels upregulated greater than 5-fold compared with the AF-treated condition) at 24 h after H1N1/H5N1 influenza virus infection **(H)** in PBMCs; Jurkat and THP1 cells; and **(I)** HEK293, HEK293T, H1650, HPF-a, and HeLa cells. **(J)** Cell viability at 24 h after H5N1 influenza virus infection was determined using MTS assay in A549, HEK293, HEK293T, HPF-a, H1650, and HeLa cells. Spearman correlation analysis of the number of TRT genes (TRT region expression levels upregulated greater than 5-fold compared with the AF-treated condition) was performed, and cell viability was calculated in H5N1-infected cell lines. Pearson correlation coefficient (r) and *P* value are provided in the graph.

Each experiment was repeated at least three times except for the RNA-seq datasets, which were established in duplicate.


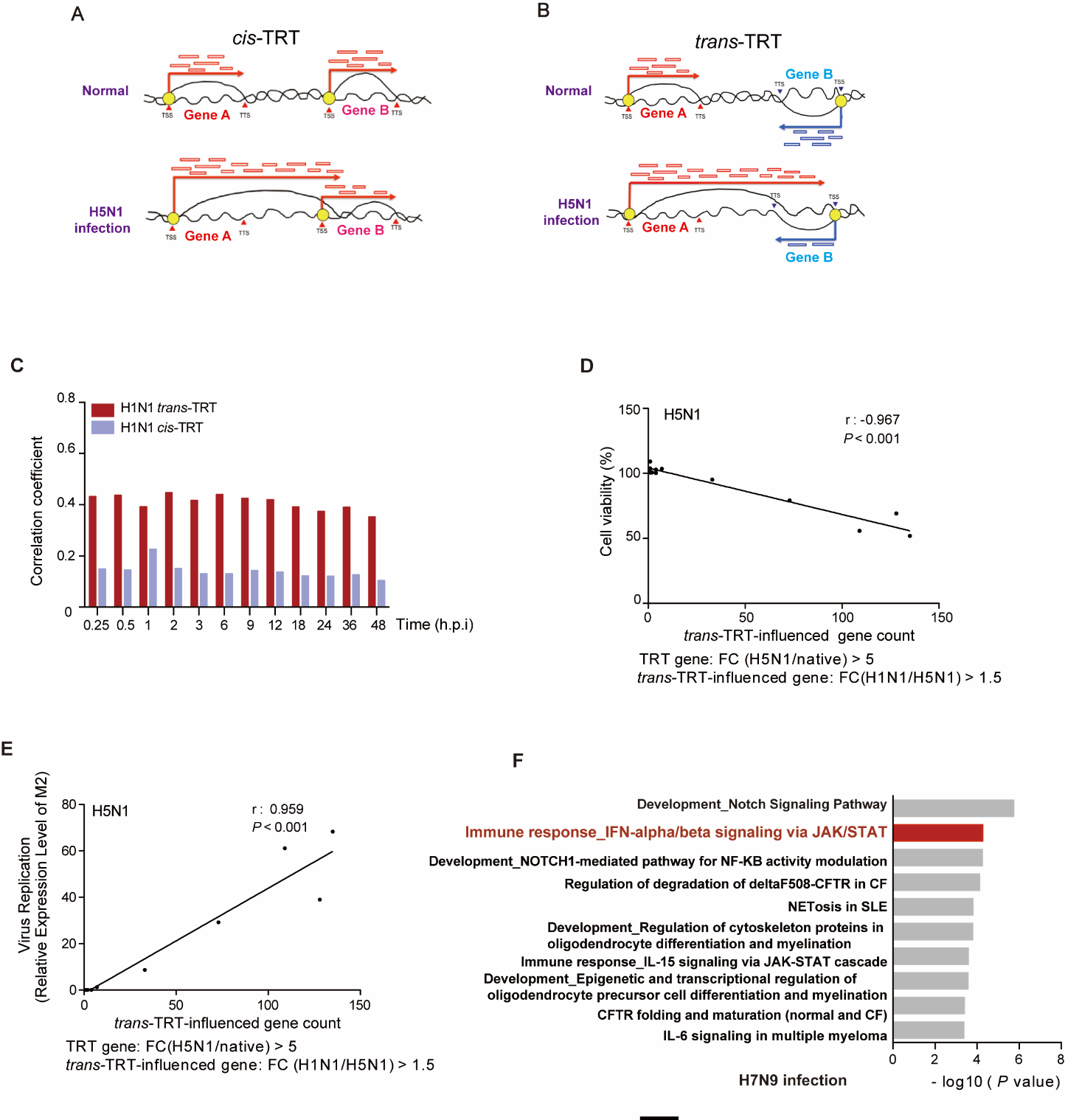


**Fig. S2 TRT represses the expression of immune response-associated genes.** **(A)** Schematic diagram of *cis*-TRT, in which TRT influences the expression of a downstream gene on the same DNA strand. **(B)** Schematic diagram of *trans*-TRT, in which the TRT of gene A influences the expression of the downstream gene B on the complementary DNA strand. **(C)** Spearman rank linear correlation analyses of the upregulated and downregulated TRT region expression levels among the *trans*-TRT/*cis*-TRT patterns of TRT-influenced genes compared with those in the AF-treated cells at 15 min, 30 min, 1 h, 2 h, 3 h, 6 h, 9 h, 12 h, 18 h, 24 h, 36 h, and 48 h after H1N1 influenza virus infection in A549 cells. **(D-E)** Correlation analysis of the numbers of *trans*-TRT-influenced genes (TRT region expression levels upregulated greater than 5-fold compared with the AF-treated condition and *trans*-TRT-influenced genes downregulated greater than 1.5-fold compared with the H1N1-infected group) and **(D)** cell viability or **(E)** virus replication (*M2*) at 0 h, 15 min, 30 min, 1 h, 2 h, 3 h, 6 h, 9 h, 12 h, 18 h, 24 h, 36 h, and 48 h after H5N1 influenza virus infection in A549 cells. A549 cell viabilities were determined using MTS assay. The H5N1 virus *M2* gene was tested using real-time PCR to determine the virus replication level. Pearson correlation coefficient (r) and *P* value are provided in the graph. **(F)** Functional pathway enrichment of *trans*-TRT-influenced genes in H7N9-infected A549 cells. A two-tailed *P* value of <0.05 and a Benjamini-adjusted *P* value of <0.05 were considered statistically significant.

RNA-seq datasets were established in duplicate.


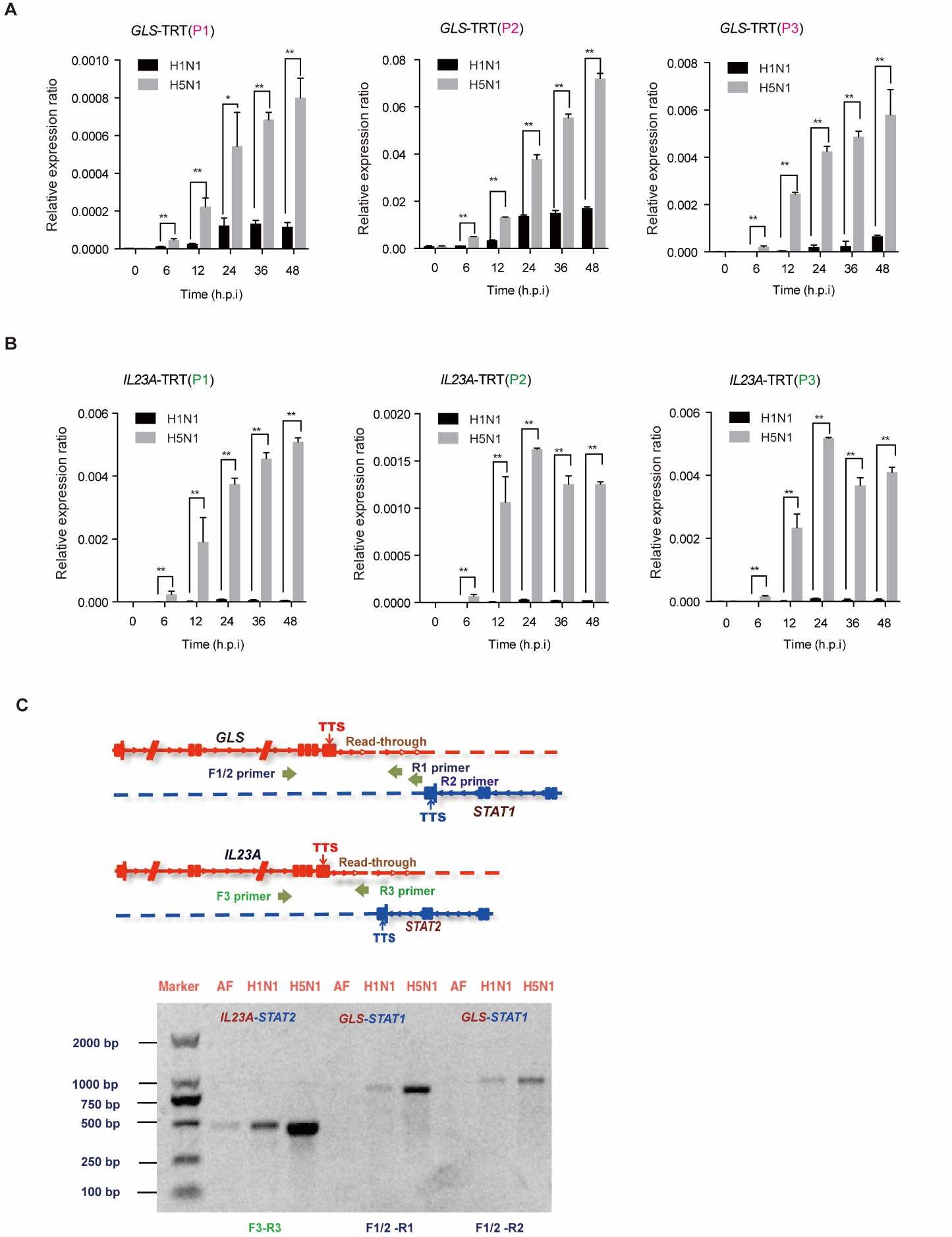


**Fig. S3 TRT could be identified using PCR after influenza virus A infection.** **(A-B)** Real-time PCR analysis of **(A)** *GLS*-TRT and **(B)** *IL23A*-TRT expression levels by using different primers (P1/P2/P3) at the indicated time points. The expression levels of *GAPDH* served as a reference control. **(C)** Schematic representation of the human genomic locus showing *GLS/IL23A* and *STAT1/STAT2* on the complementary DNA strand. Red and blue boxes represent exons. Polyadenylation (pA) sites mark the transcription termination sites. Different pairs of primers represent the detection of the intermediate transcripts by PCR. The primer sequences are listed in Supplementary Table 1.

Each experiment was repeated at least three times.

The data are shown as the means ± SEMs. **P* < 0.05, ***P* < 0.01, and ****P* < 0.001.


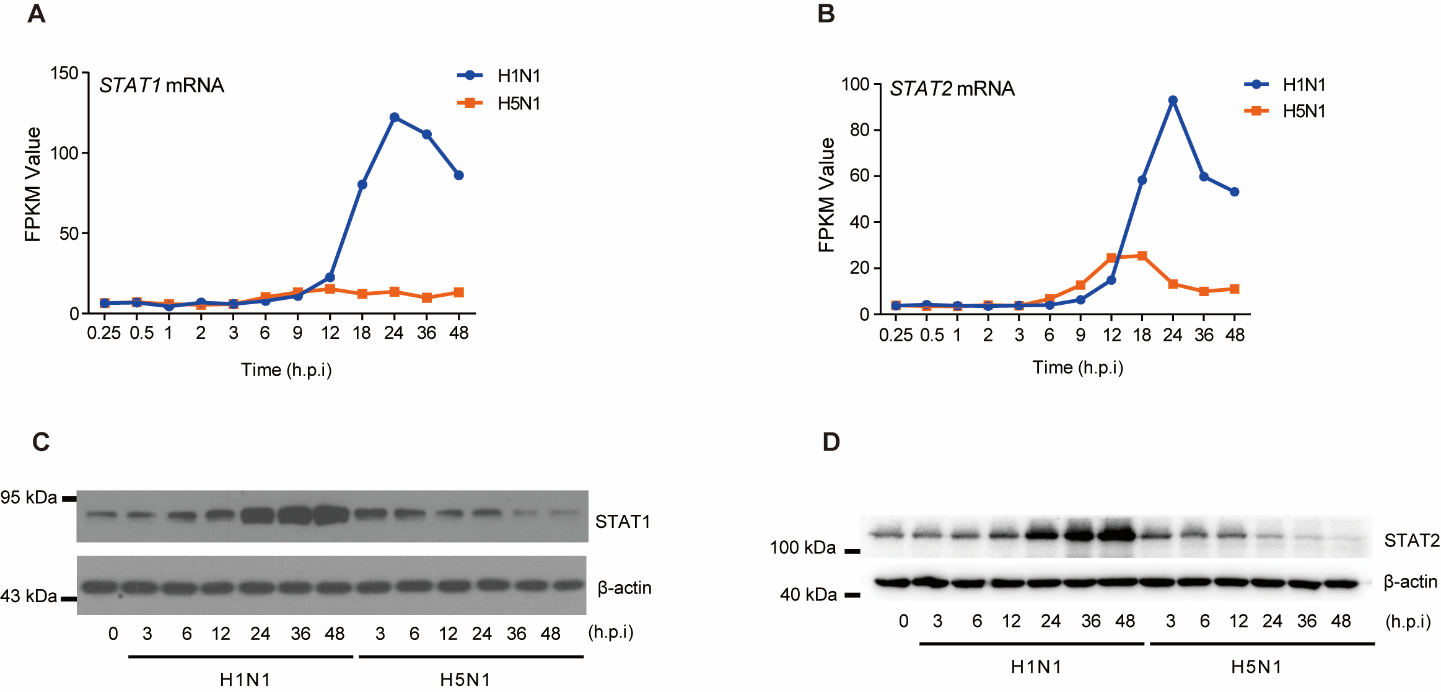


**Fig. S4 TRT represses the expression of complementary strand genes such as *STAT1/STAT2.* (A-B)** FPKM values of the **(A)** *STAT1* and **(B)** *STAT2* genes in A549 cells infected with the H1N1 virus (MOI = 4) or H5N1 virus (MOI = 4) for 15 min, 30 min, 1 h, 2 h, 3 h, 6 h, 9 h, 12 h, 18 h, 24 h, 36 h, and 48 h. **(C-D)** Western blot analysis of **(C)** STAT1 and **(D)** STAT2 at the indicated time points. The expression levels of β-actin served as a reference control.

The RNA-seq datasets were established in duplicate.

Each experiment was repeated at least three times.


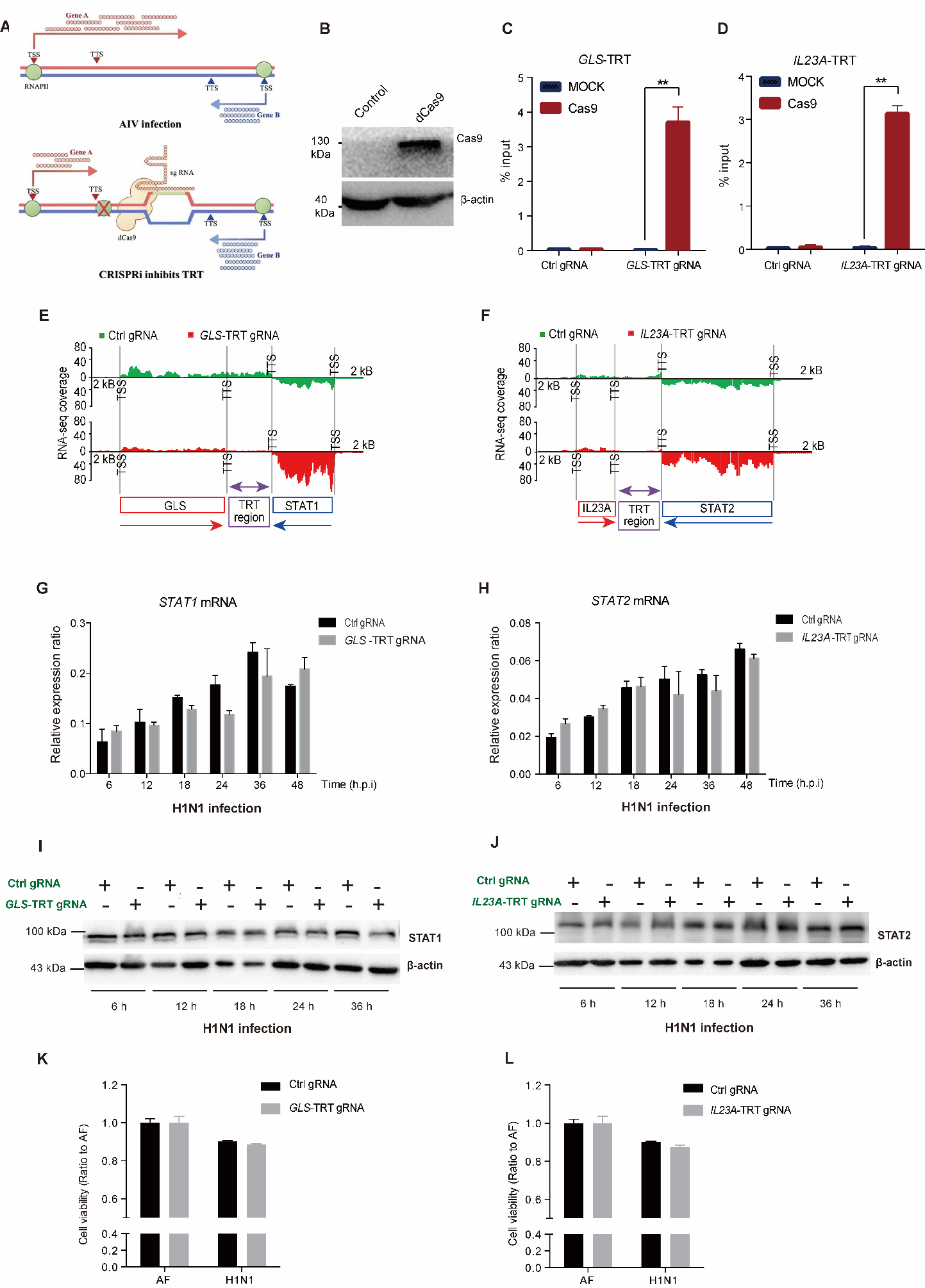


**Fig. S5 TRT inhibition by CRISPRi could not improve *STAT1/STAT2* expression after H1N1 infection. (A)** Schematic representation of the CRISPRi technique used to suppress TRT. **(B)** Western blot analysis of dCas9 in control A549 cells and in dCas9 overexpression monoclonal A549 cells (β-actin expression served as a reference control). **(C-D),** Chromatin immunoprecipitation–quantitative PCR (ChIP-qPCR) was performed using an antibody directed against Cas9 and a control IgG (MOCK) in A549 cells stably expressing dCas9. Quantitative PCR was performed using primers specific for the **(C)** *GLS*-TRT or **(D)** *IL23A*-TRT target region. Control gRNA was used as a negative control. The figure shows the recovery expressed as the percentage of input (relative amount of immunoprecipitated DNA compared with the input DNA after qPCR analysis). **(E-F)** RNA-seq coverage levels of (**E**) the *GLS* gene, TRT region of the *GLS* gene, and *GLS* gene *trans*-TRT-influenced *STAT1* gene in Ctrl gRNA and *GLS*-TRT gRNA groups and (**F**) the *IL23A* gene, TRT region of the *IL23A* gene, and *IL23A* gene *trans*-TRT-influenced *STAT2* gene in Ctrl gRNA and *GLS*-TRT gRNA groups at 24 h after H5N1 infection. Gene body and intergenic regions, as well as gene-flanking regions 2 Kb upstream of the TSSs, were spliced into 50-bp windows. Only exon regions of the *GLS, STAT1, IL-23A,* and *STAT2* genes are shown in this graph. **(G-H)** Real-time PCR analysis of **(G)** *STAT1* and **(H)** *STAT2* mRNA expression in the Ctrl gRNA and *GLS*-TRT/*IL23A*-TRT gRNA groups at 6 h, 12 h, 18 h, 24 h, 36 h, and 48 h after infection with H1N1 (MOI = 4). **(I-J)** Western blot analysis of the **(I)** STAT1 protein expression in the Ctrl gRNA and *GLS*-TRT gRNA groups and the **(J)** STAT2 protein expression in the Ctrl gRNA and *IL23A*-TRT gRNA groups at 6 h, 12 h, 18 h, 24 h, and 36 h after infection with H1N1 (MOI = 4). The relative expression density was analysed using ImageJ, and β-actin served as a reference control. **(K-L)** MTS analysis of the cell viability of the **(K)** *GLS*-TRT gRNA and **(L)** *IL23A*-TRT gRNA groups after 48 h of treatment with AF or 48 h of infection with the H1N1 virus (MOI = 4).

The data are shown as the means ± SEMs. **P* < 0.05, ***P* < 0.01.

Each experiment was repeated at least three times.


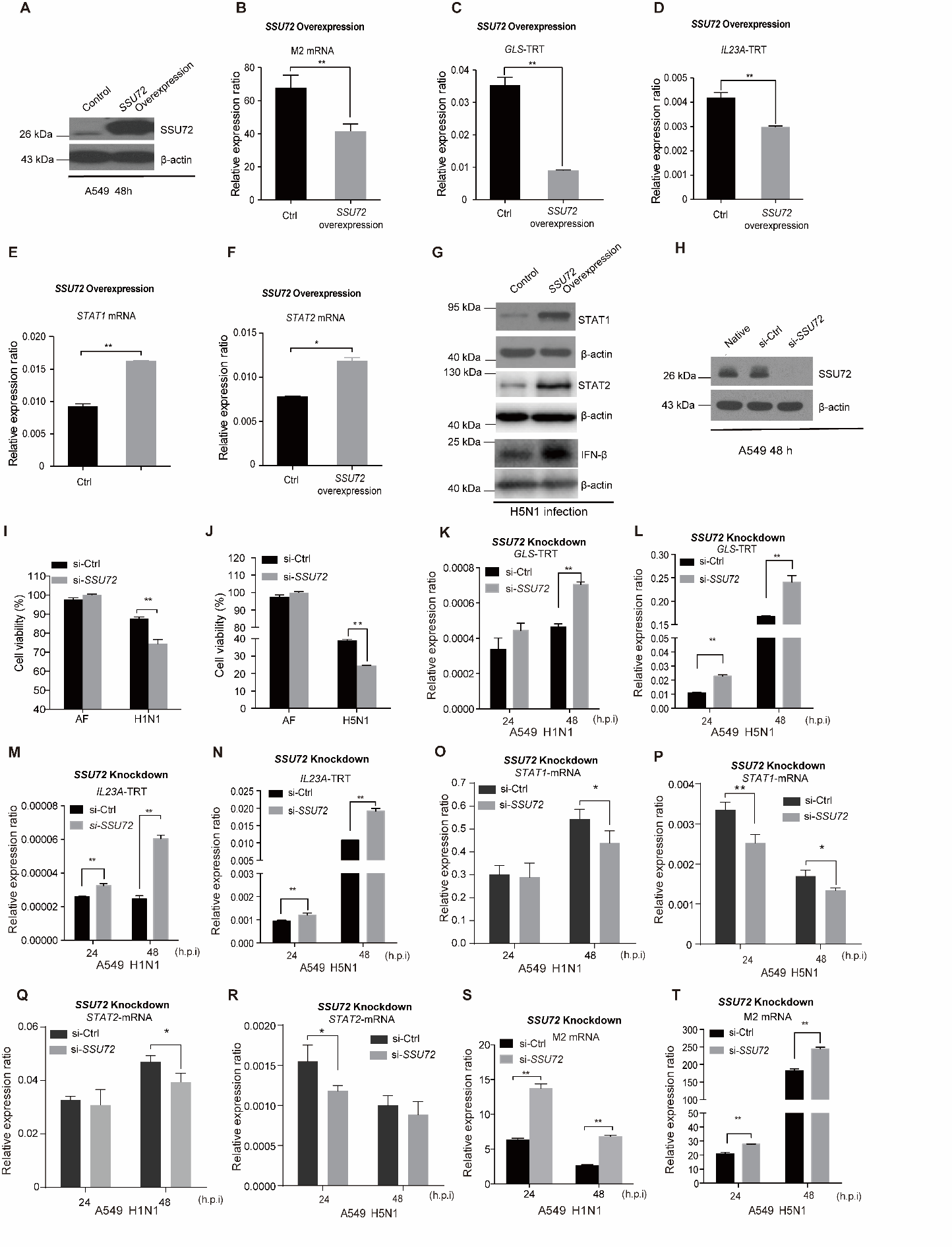


**Fig. S6 Gain or loss of SSU72 function influences TRT. (A-G)** A549 cells were transfected with an SSU72 overexpression plasmid or a control plasmid for 60 h and then treated with AF or infected with H5N1 (MOI = 4). **(A)** Western blot analysis of the SSU72 protein expression levels at 48 h after transfection with the control or SSU72 overexpression plasmid in A549 cells. The expression levels of β-actin served as a reference control. **(B)** Real-time PCR analysis of the relative expression levels of influenza virus M2 mRNA at 24 h after H5N1 influenza virus infection. The expression levels of *GAPDH* served as a reference control. **(C-D)** Real-time PCR analysis of **(C)** *GLS*-TRT and **(D)** *IL23A*-TRT at 24 h after H5N1 influenza virus infection. The expression levels of *GAPDH* served as a reference control. **(E-F)** Real-time PCR analysis of **(E)** *STAT1* and **(F)** *STAT2* mRNA at the indicated time points. The expression levels of *GAPDH* served as a reference control. **(G)** Western blot analysis of STAT1, STAT2, and IFN-β at 24 h after H5N1 influenza virus infection. The expression levels of β-actin served as a reference control. **(H-P)** A549 cells were transfected with a control siRNA or an *SSU72*-specific siRNA for 48 h and then treated with AF/H1N1/H5N1. **(H)** Western blot analysis of the SSU72 protein expression levels at 48 h after transfection with the control siRNA or *SSU72*-specific siRNA in A549 cells. The expression levels of β-actin served as a reference control. **(I-J),** MTS analysis of the viability of control cells and *SSU72* knockdown cells at 48 h after H1N1 **(I)** and H5N1 **(J)** virus infection. **(K-N)** Real-time PCR analysis of *GLS*-TRT of control cells and *SSU72* knockdown cells at 24 h and 48 h after H1N1 **(K)** and H5N1 **(L)** influenza virus infection. Real-time PCR analysis of *IL-23A*-TRT of control cells and *SSU72* knockdown cells at 24 h and 48 h after H1N1 **(M)** and H5N1 **(N)** influenza virus infection. The expression levels of *GAPDH* served as a reference control. **(O-R)** Real-time PCR analysis of *STAT1* mRNA of control cells and *SSU72* knockdown cells at 24 h and 48 h after H1N1 **(O)** and H5N1 **(P)** influenza virus infection. Real-time PCR analysis of *STAT2* mRNA of control cells and *SSU72* knockdown cells at 24 h and 48 h after H1N1 **(Q)** and H5N1 **(R)** influenza virus infection. **(S-T)** Real-time PCR analysis of the relative expression levels of influenza virus *M2* mRNA of control cells and *SSU72* knockdown cells at 24 h and 48 h after H1N1 **(Q)** and H5N1 **(R)** influenza virus infection. The expression levels of *GAPDH* served as the reference control.

Each experiment was repeated at least three times.

The data are shown as the means ± SEMs. **P* < 0.05, ***P* < 0.01.


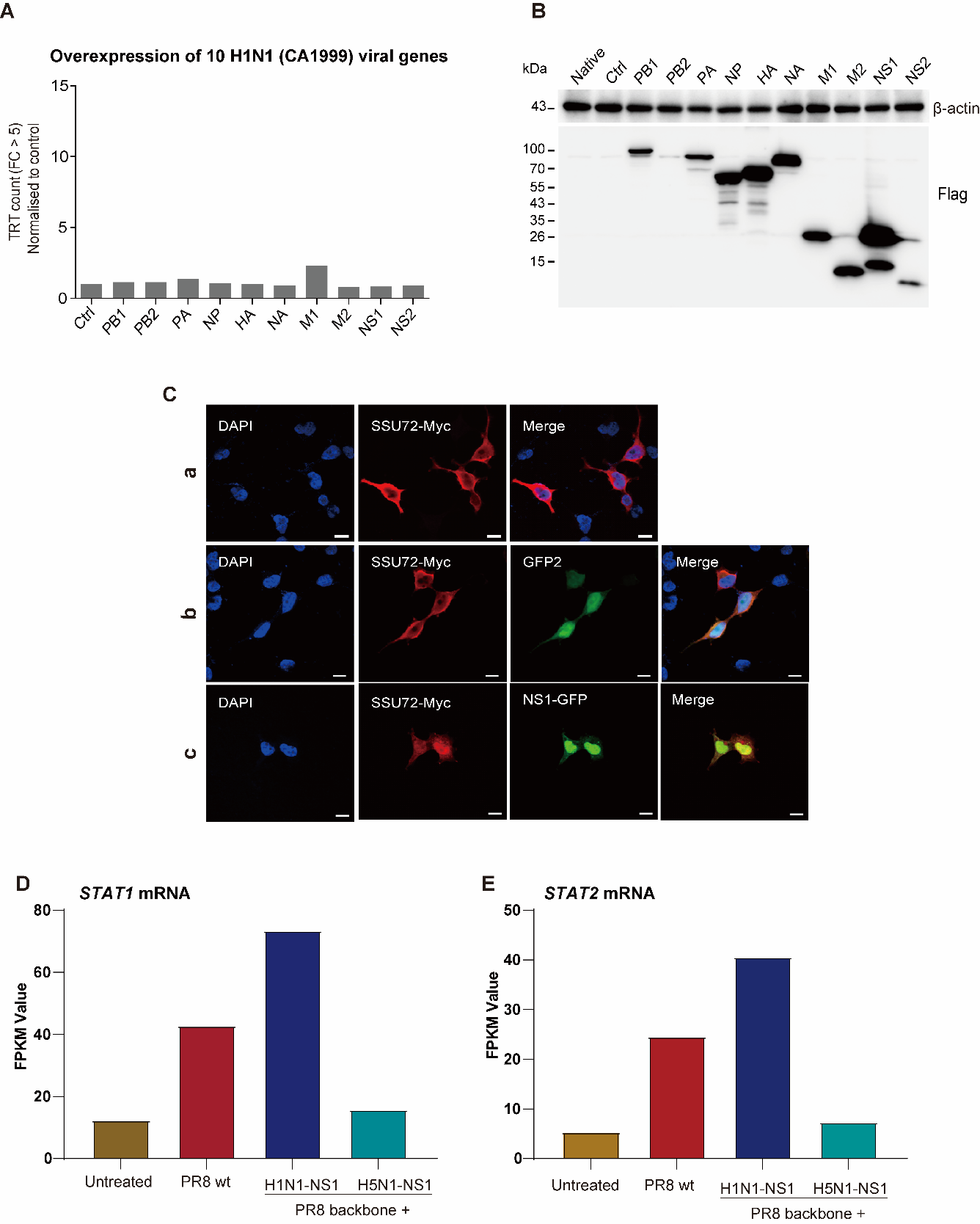


**Fig. S7 SSU72 binds to NS1 of the H5N1 virus. (A)** Numbers of TRT genes (TRT region expression levels upregulated greater than 5-fold compared with the control plasmid condition) in the A/New Caledonia/20/1999 (H1N1) virus gene segments *PB1*, *PB2*, *PA*, *NP*, *HA*, *NA*, *M1*, *M2*, *NS1*, and *NS2* in the vector plasmid groups in HEK239T cells. **(B)** Western blot analysis of Flag tag expression at 48 h after overexpression of 10 H1N1 plasmids (β-actin expression served as a reference control). **(C)** Confocal immunofluorescence images of HEK293T cells cotransfected with SSU72 and different plasmids. (a) SSU72-Myc only; (b) SSU72-Myc and GFP; and (c) SSU72-Myc and H5N1 NS1-GFP. Images were obtained 48 h after transfection. Slides were stained with an anti-Myc antibody (red) and DAPI (blue) to visualise nuclei. **(D-E)** FPKM values of *STAT1* **(D)** and *STAT2* **(E)** genes in A549 cells by virus untreated and treated by PR8 wildtype virus and PR8 backbone recombined with NS1 of the A/New Caledonia/20/1999(H1N1) or NS1 of A/Chicken/Jilin/9/2004(H5N1) recombinant influenza virus.

The RNA-seq datasets were established once. The confocal immunofluorescence experiment was repeated at least three times.


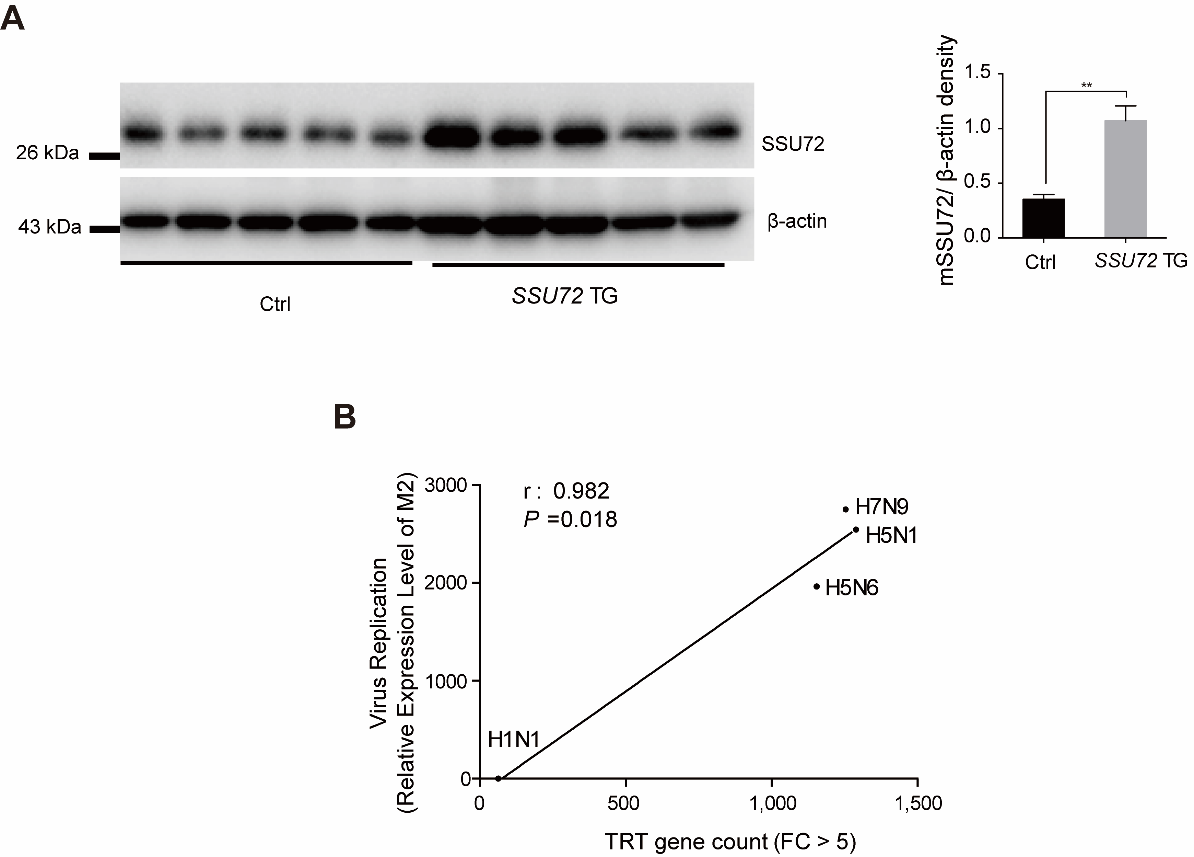


**Fig. S8 The SSU72 expression in Control and *SSU72* transgenic mice and TRT correlates with virus replication. (A)**Western blot analysis of the SSU72 protein expression levels in whole lungs from control (n = 5) and *SSU72* transgenic (n = 5) mice. β-actin expression served as an internal control. **(B)** Correlation analysis of the number of TRT genes (TRT region expression levels upregulated with an FC greater than 5 compared with the AF-treated condition) and virus replication at 48 h after H1N1/H5N1/H5N6/H7N9 influenza virus infection in A549 cells. The expression level of the influenza virus M2 gene was analysed using real-time PCR to indicate the virus replication level. Pearson correlation coefficient (r) and *P* value are provided in the graph.

Each experiment was repeated at least three times.

The data are shown as the means ± SEMs. **P* < 0.05, ***P* < 0.01.

**Supplementary Tables**

**Table S1.** Probes and Primers of PCR Analysis.

| Gene Name | Primer Sequence (5'-3') |
| --- | --- |
| *GLS*-TRT Probe | AAGCGAATGCTATTCCCACG |
| *IL23A*-TRT Probe | GCTGATTGTTGGCAAAGCC |
| human *GAPDH* mRNA-Forward | CGGAGTCAACGGATTTGGTC |
| human *GAPDH* mRNA-Reverse | TGGGTGGAATCATATTGGAACAT |
| human *STAT1* mRNA-Forward | CTACAGCATAACATAAGGA |
| human *STAT1* mRNA-Reverse | CATAGACATCTGGATTGG |
| human *STAT2* mRNA-Forward | GGCAGTTCTCCTCCTATG |
| human *STAT2* mRNA-Reverse | TCCTCAGTCCTACAGTTCT |
| human *SSU72* mRNA-Forward | AAGAACTCTATACACAGAATGG |
| human *SSU72* mRNA-Reverse | CACCTGGTCATACACTCT |
| *GLS*-TRT (P1) Forward | GCATTACTGGCATTGTAGAA |
| *GLS*-TRT (P1) Reverse | AGAAGAGTGAGTTAGATTAGAGAA |
| *GLS*-TRT (P2) Forward | ATCCCACTGATGATGTAG |
| *GLS*-TRT (P2) Reverse | CAGGGAGCGCAGGATGG |
| *GLS*-TRT (P3) Forward | TACGAAGATATGGAGATGAGAATA |
| *GLS*-TRT (P3) Reverse | AGGCATAGTCTGTCAAGTT |
| *IL23A*-TRT (P1) Forward | ACTGTCTCATTGTCACTCA |
| *IL23A*-TRT (P1) Reverse | TCCATAGTCACAGCATCAA |
| *IL23A*-TRT (P2) Forward | TGATGCTGTGACTATGGAA |
| *IL23A*-TRT (P2) Reverse | GTTTGGTAACTCAAGGGTAG |
| *IL23A*-TRT (P3) Forward | TTCCATAGTCACAGCATCA |
| *IL23A*-TRT (P3) Reverse | ACTGTCTCATTGTCACTCA |
| *GLS*-*STAT1* Forward -1/2 | GGACTGAATGACCTGATGT |
| *GLS*-*STAT1* Reverse-1 | AGTTCTACAATGCCAGTAATG |
| *GLS*-*STAT1* Reverse-2 | AGAAGAGTGAGTTAGATTAGAGAA |
| *IL23A*-*STAT2* Forward | GCCAAGAGACTACAATATGC |
| *IL23A*-STAT2 Reverse | CAGGAGAATCGCTTCAAC |
| *GLS*-TRT gRNA Forward for ChIP | ATGAGCCAGGTACTATGC |
| *GLS*-TRT gRNA Reverse for ChIP | GGCATAATCAGTGTTGTAGG |
| *IL23A*-TRT gRNA Forward for ChIP | GCATCCCTTTCTTCTATTCTG |
| *IL23A*-TRT gRNA Reverse for ChIP | GGTATGATCCCACTTCCTAA |
| *M2* mRNA-Forward | ATTGTGGATTCTTGATCGTC |
| *M2* mRNA-Reverse | TGACAAAATGACCATCGTC |
| mouse *β-actin* mRNA-Forward | CTCTCCCTCACGCCATCC |
| mouse *β-actin* mRNA-Reverse | CGCACGATTTCCCTCTCAG |
| mouse *STAT1* mRNA-Forward | CGAACTGGATACATCAAGAC |
| mouse *STAT1* mRNA-Reverse | TTATACTGTGCTCATCATACTG |
| mouse *STAT2* mRNA-Forward | CACTCCGCTTCCTCTATC |
| mouse *STAT2* mRNA-Reverse | CGTCCACCTGTCTGTTAG |

**Table S2.** Characteristics of Patients with Influenza A Virus Infection, Pneumonia Patients and Healthy Controls

| Characteristics | Value | | |
| --- | --- | --- | --- |
| Healthy controls-no. | 10 | | |
| Age |  | | |
| Mean(SD)-yr | 29.11(4.20) | | |
| P^a^ value | 0.009 | | |
| P^b^ value | 0.018 | | |
| P^c^ value | 0.001 | | |
| P^d^ value | 0.396 | | |
| Female sex–no.(%) | 5(50) | | |
| P^a^ value | 0.664 | | |
| P^b^ value | 0.397 | | |
| P^c^ value | 0.378 | | |
| P^d^ value | 0.612 | | |
| Pneumonia patients-no. | 8 | | |
| Age |  | | |
| Mean(SD)-yr | 45.38(15.74) | | |
| P^e^ value | 0.262 | | |
| Female sex–no.(%) | 3(37.5) | | |
| P^e^ value | 0.642 | | |
| H1N1 patients-no. | 15 | | |
| Age |  | | |
| Mean(SD)-yr | 55.27(24.53) | | |
| P^f^ value | 0.696 | | |
| Female sex–no.(%) | 4(26.67) | | |
| P^f^ value | 0.922 | | |
| H7N9 patients-no. | 12 | | |
| Age |  | | |
| Mean(SD)-yr | 53.58(15.38) | | |
| Subgroup-no.(%) |  | | |
| ≤65 | 9(75) | | |
| >65 | 3(25) | | |
| Female sex–no.(%) | 3(25) | | |
| Coexisting conditions–no.(%) |  | | |
| Hypertension | 4(33.3) | | |
| Pathoglycemia | 2(16.67) | | |
| Dyslipidemia | 2(16.67) | | |
| Diabetes | 1(8.33) | | |
| Coronary heart disease | 1(8.33) | | |
| Rheumatic heart disease | 1(8.33) | | |
| Arrhythmia | 1(8.33) | | |
| Fatty liver | 1(8.33) | | |
| Hepatitis | 1(8.33) | | |
| Chronic renal insufficiency | 1(8.33) | | |
| Lumbar disc herniation | 1(8.33) | | |
| Treatment-no.(%) |  | | |
| Administration of oseltamivir | 12(100) | | |
| PBMC sampling-no.(%) |  | | |
| the first week of illness onset | 4(33.3) | | |
| the second week of illness onset | 11(91.67) | | |
| after two weeks of illness onset | 11(91.67) | | |
| Clinical outcome |  | | |
| Death–no.(%) | 3(25) | | |
| Discharge from hospital–no.(%) | 9(75) | | |
| H5N6 patients-no. | | 3 | |
| Age | |  | |
| Mean(SD)-yr | | 27.33(3.21) | |
| Female sex–no.(%) | | 2(66.7) | |
| Treatment-no.(%) | |  | |
| Administration of oseltamivir | | 3(100) | |
| Clinical outcome | |  | |
| Death–no.(%) | | 2(66.7) | |
| Discharge from hospital–no.(%) | | 1(33.3) | |
| PBMC: peripheral blood mononuclear cell  P^a^ value: Healthy controls vs Pneumonia patients group  P^b^ value: Healthy controls vs H1N1 patients group  P^c^ value: Healthy controls vs H7N9 patients group  P^d^ value: Healthy controls vs H5N6 patients group  P^e^ value: Pneumonia patients group vs H7N9 patients group  P^f^ value: H1N1 patients group vs H7N9 patients group | | |  |
